# Chiral nonlinear polaritonics with van der Waals metasurfaces

**DOI:** 10.1126/sciadv.aeb5631

**Published:** 2026-03-27

**Authors:** Connor Heimig, Alexander A. Antonov, Dmytro Gryb, Thomas Possmayer, Thomas Weber, Michael Hirler, Jonas Biechteler, Luca Sortino, Leonardo de S. Menezes, Stefan A. Maier, Maxim V. Gorkunov, Yuri Kivshar, Andreas Tittl

**Affiliations:** ^1^ Chair in Hybrid Nanosystems, Nanoinstitute Munich, Faculty of Physics, Ludwig-Maximilians-Universität München, Munich 80539, Germany.; ^2^ Departamento de Física, Universidade Federal de Pernambuco, Recife-PE 50670-901, Brazil.; ^3^ School of Physics and Astronomy, Monash University, Clayton, Victoria, VIC 3168, Australia.; ^4^ Department of Physics, Imperial College London, London SW7 2BW, UK.; ^5^ Shubnikov Institute of Crystallography, National Research Centre, Kurchatov Institute, Moscow 119333, Russia.; ^6^ Theoretical Physics and Quantum Technologies Department, National University of Science and Technology “MISIS,” Moscow 119049, Russia.; ^7^ Nonlinear Physics Centre, Research School of Physics, Australian National University, Canberra, ACT 2601, Australia.

## Abstract

Chiral optical cavities are crucial for the development of nonequilibrium quantum materials by discriminating and selectively coupling to light of a specific circular polarization, but fundamentally cannot be realized with conventional mirror cavities. Here, we demonstrate this unique functionality by developing a monolithic transition metal dichalcogenide (TMDC) metasurface with broken out-of-plane symmetry, allowing for the selective formation of self-hybridized chiral exciton-polaritons. Our metasurface maintains maximal chirality for oblique incidence up to 20°, thereby outperforming all previously known designs. Moreover, we study the chiral strong-coupling regime in nonlinear experiments and reveal polaritonic signatures in chiral third-harmonic generation. Our results position maximally chiral van der Waals (vdW) metasurfaces as a versatile platform for tunable chiral polaritonics with applications in nonreciprocal photonic devices and valleytronics.

## Teaser

A maximally chiral metasurface enables polariton-driven third-harmonic generation with controllable light helicity.

## INTRODUCTION

The interaction of chiral light and matter has become a notable area of interest within the broader nanophotonics community, including chiral sensing (*1*, *2*), quantum emission (*3*), and photonic circuitry (*4*). Recently, the emerging class of two-dimensional (2D) materials has become a focal point for these investigations, with a particular emphasis on the family of transition metal dichalcogenides (TMDCs) (*5*, *6*). The role of chirality is especially prominent in TMDC research because it plays a crucial role in the formation of excitonic valleys in TMDC monolayers (*7*). These atomically layered materials exhibit considerable application potential due to their electronic and optical properties, which have been used in a diverse range of research areas, including spintronics (*8*), electrocatalysis (*9*), topological insulators (*10*), and superconductivity (*11*).

A defining feature of these materials is their pronounced excitonic properties, particularly when examined in the context of strong coupling (*12*, *13*). This denotes a condition wherein the interaction between light and matter becomes so intense that they can no longer be regarded as independent entities. Instead, they give rise to hybrid quasi-particles, known as polaritons, exhibiting attributes from both underlying components (*14*). These hybrid properties bring about a host of intriguing phenomena, with a wide range of applications from quantum computing (*15*) to lasing (*16*). To achieve strong coupling, a frequent approach is to place an excitonic system within or in proximity to an optical cavity, which provides the requisite confined light mode (*17*). Chiral polaritonics has the potential to become a new frontier for nanophotonics (*18*), with first successful experimental realizations based on chiral plasmonic-excitonic systems (*19*) and DNA origami (*20*). Previous theoretical investigations provided initial insights into the chiral strong-coupling picture by combining material chirality with excitons (*21*). The impact of a chiral cavity on graphene was also studied theoretically, displaying the quantized light-induced anomalous Hall effect (*22*). Still, the experimental development of more generally applicable chiral polaritonic platforms that move beyond material-intrinsic chirality remains a crucial and persistent need.

Combining conventional optical resonators, such as mirror-based Fabry-Pérot cavities, with excitonic materials enables strong coupling between photonic modes and excitons (*23*). However, generating the chiral optical fields necessary for chiral strong coupling typically requires elaborate configurations, such as precisely aligned pairs of chiral mirrors (*24*, *25*). Here, we propose an alternative approach: a single metasurface that simultaneously supports a high-quality-factor chiral optical resonance and incorporates a TMDC material with strong excitonic response. Metasurfaces are artificially nanoengineered planar structures that have been specifically designed to manipulate light by periodically arraying subwavelength building blocks, enabling a wide range of optical functionalities (*26*–*28*). When aiming to confine a light mode in a metasurface, the concept of bound states in the continuum (BIC) has emerged as a promising candidate (*29*). This allows precise control over the radiative losses in the system as well as the confinement of ultrasharp resonances. Such BIC metasurfaces serve the same function in polaritonics as a cavity, providing the near-field enhancement necessary to strongly couple light with an exciton. In this way, they function analogously to open cavity systems but in a simpler and more compact form, as strong coupling occurs within a much smaller volume and does not require opposing reflectors. Furthermore, metasurfaces have been shown to support maximally chiral eigenstates that are uncoupled from light of a particular circular polarization while resonantly interacting with its opposite-handed counterpart (*30*, *31*).

Previous work aimed at coupling excitons in TMDCs to a confined BIC mode was based on the transfer or growth of a single atomic layer onto a metasurface fabricated from conventional dielectric materials (*32*, *33*). This approach suffers from numerous inherent limitations, from scalability to strain-driven alterations to the atomic layers’ properties (*34*). The principal benefit of our monolithic methodology (where the entire metasurface is fabricated from bulk TMDC) is that it enables the investigation of intrinsic characteristics, such as the nonlinearities inherent to this class of materials. Moreover, understanding of the nonlinear response of bulk TMDCs potentially facilitates a multitude of technological applications, as nonlinear optical processes are a fundamental aspect of commercially available frequency mixing and conversion or lasing systems (*35*, *36*). Harmonic generation offers vast research potential, exemplified by applications such as biosensing (*37*), microscopy (*38*), and, more generally, the ability to generate optical pulses varying on attosecond timescales, enabling the study of ultrafast processes with unprecedented temporal and spatial resolution (*39*).

Here, we experimentally demonstrate chiral light-matter coupling by merging maximally chiral metasurfaces with excitons in van der Waals (vdW) semiconductors. We develop and experimentally realize a monolithic WS_2_ metasurface with out-of-plane symmetry breaking, simultaneously addressing two fundamental constraints of cavity physics. First, our approach allows for facile and substantial tuning of the metasurface chiral resonance via the incidence angle, going beyond the established invasive and irreversible cavity tuning via stimuli-responsive materials (*40*). Crucially, our tuning mechanism maintains full system functionality (i.e., resonance modulation and maximal chirality) throughout the tuning range, greatly exceeding previous approaches (*41*). Second, we experimentally realize a chiral polaritonic system using our vdW metasurface as an open cavity, showing clear handedness-selective anticrossing behavior and Rabi splitting via self-hybridization of chiral exciton-polaritons. Furthermore, we use this system to implement the theoretically predicted but so far experimentally elusive chiral polaritonic third-harmonic emission in TMDCs.

## RESULTS

### Maximally chiral metasurfaces for self-hybridized chiral exciton-polaritons

**Fig. 1.**
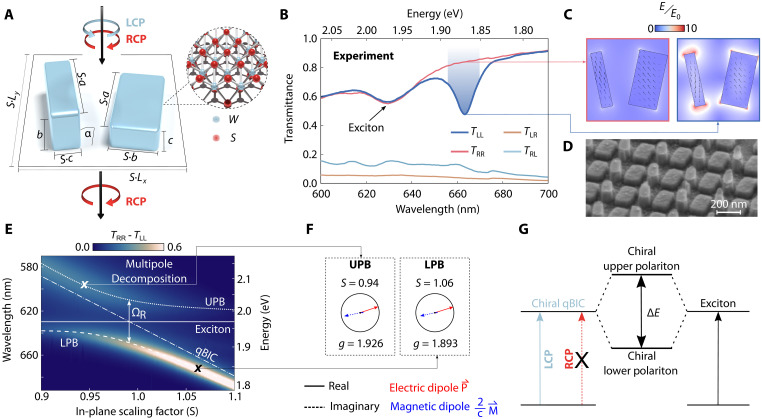
Maximally chiral vdW metasurfaces for strong coupling. (**A**) Out-of-plane symmetry breaking enables chiral qBIC resonances in 3D monolithic WS_2_ metasurfaces. The unit cell design has a periodicity of *L_x_* = *L_y_* = 375 nm, with rod side lengths of *a* = 225 nm, *b* = 115 nm, and *c* = 45 nm, respectively. The height difference is ∆*h* = *b* − *c* = 70 nm. Each rod is rotated by an angle of α = 12°. (**B**) Experimental co-/cross-polarized chiral transmittance spectra verify maximal chirality, showing the selective emergence of a qBIC resonance in the left copolarized signal (this panel uses a slightly different layout *L_x_* = *L_y_* = 385 nm, α = 9°, *a* = 220 nm, *b* = 60 nm and 135 nm, Δ*h* = 50 nm, *h* = 85 nm, and *S* = 1.1). (**C**) Simulated electric near-field enhancement (*z* = 80 nm, i.e., average height value of the rods) at the qBIC resonance for a left-handed structure under LCP/RCP illumination. (**D**) SEM image of the fabricated chiral metasurface on a SiO_2_ substrate. (**E**) Simulated transmittance difference ∆*T* = *T*_RR_ − *T*_LL_ of the metasurface for different in-plane scaling factors *S* shows the emergence of lower (LPB) and upper (UPB) polaritonic branches with anticrossing behavior. (**F**) Multipole decomposition analysis of the eigenstates of two different scaling factors, one from each of the respective polariton branches. The dissymmetry factor *g* (see Eq. 1) reaches values close to the theoretical upper limit *g*_max_ = 2 for both cases. (**G**) Simplified energy-level diagram of self-hybridization of excitons and qBIC into chiral polaritons.

Our metasurface design adopts a rod-type unit cell geometry with broken out-of-plane symmetry, where each unit cell contains two identical rods lying on different facets (Fig. 1, A and D). By controlling the opening angle α between the rods and their height difference ∆*h*, the antiparallel electric dipole BIC is transformed into a radiative maximally chiral quasi-BIC (qBIC) (see note S1). This approach has previously been shown for chiral metasurfaces at microwave wavelengths using ceramic resonators (*42*) and close to the red part of the visible spectrum using silicon (*43*), but has so far not been brought to experiments involving vdW materials and wavelengths in the visible range. In our metasurface structure, the resonators are composed solely of bulk WS_2_ to enable self-hybridized light-matter coupling (*44*). To realize such structures experimentally, a fabrication workflow relying on a multistep top-down nanofabrication process had to be developed, merging both inverse and lift-off techniques. Notably, this method represents a notable improvement over previous approaches because it is not limited to the structuring of sputtered/evaporated materials, thus offering general applicability as evidenced by our work with exfoliated materials, while also achieving superior accuracy (for fabrication details see note S3).

The qBIC resonance can be spectrally shifted by continuously varying the geometric parameters of the structure. However, since the height of a single flake cannot be continuously tuned, we use an in-plane scaling factor *S*, which scales the structures only in the *xy*-plane. Throughout this work, we use left-handed metasurface structures, where the qBIC resonance is uncoupled from right circularly polarized (RCP) light. We observe maximal chirality in experimental transmittance measurements, characterized by negligible cross-polarized signals *T*_RL_ and *T*_LR_ and selective mode formation in the copolarized signal *T*_LL_ (Fig. 1B). Here, the indices *f* and *i* in *T_fi_* correspond to the final and initial polarization states, respectively.

The qBIC-driven metasurface concept enables direct control over the radiative quality (*Q*) factor of the chiral qBIC via introducing the perturbation parameters: opening angle α and height difference ∆*h* (*41*). However, an upper limit on the achievable *Q* factor is imposed by material-intrinsic loss channels, as well as other parasitic losses such as fabrication imperfections, surface roughness, or edge effects (*45*). The perturbation parameters α and ∆*h* have a direct impact on the chiral response, which approaches maximal chirality at α ∼ *k*∆*h* (with *k* being the free-space light wave number; see Supplementary Note 1) (*42*). Previous work has shown that the Rabi splitting in the strong coupling regime is maximized when the total damping rates (of the exciton γ_Ex_ and qBIC γ_qBIC_, respectively) in the system are matched (*44*). The splitting decreases as γ_qBIC_ moves away from this matching condition, more steeply when the qBIC is broader than the exciton and more gradually when it is narrower. Although lowering the qBIC *Q* factor might be expected to suppress the splitting via a transition to weak coupling, note S8 shows that the strong-coupling condition holds for all realistic, tabulated exciton oscillator strengths; weak coupling is therefore unattainable in our system.

Higher *Q* maximally chiral resonances could be achieved by simultaneously decreasing the opening angle α and the height difference Δ*h*; doing so shifts the system away from linewidth matching, reduces the observed Rabi splitting, and renders the polaritonic resonances, particularly the UPB, harder to resolve due to higher intrinsic material losses. Consequently, we deliberately selected resonances with total linewidths comparable to that of the exciton for the experiment (α = 12° and Δ*h* = 70 nm), which allowed us to resolve the UPB while maximizing the observed Rabi splitting.

The simulated electric near field at resonance exhibits no enhancement for RCP excitation, whereas a 10-fold enhancement is observed for left circularly polarized (LCP) excitation, verifying the formation of a maximally chiral mode (Fig. 1C). By varying the geometry of the metasurface through the in-plane scaling factor *S*, the qBIC resonance wavelength can be shifted across the spectral range of the room temperature exciton in WS_2_ at 629 nm (1.971 eV). The resulting transmittance difference ∆*T* = *T*_RR_ − *T*_LL_ exhibits a characteristic anticrossing pattern around the exciton position (Fig. 1E). Note that ∆*T* highlights absolute resonance contrast (sensitive to material absorption) rather than intrinsic chirality.

To assess the chirality of the resulting exciton-polaritons, we numerically perform multipole decomposition of the hybrid eigenstates. Specifically, we decompose the coupling coefficients between the displacement current associated with the polaritonic eigenstate and free-space plane waves for metasurfaces with a given geometric scaling. The computed dipole moments of these eigenstates (Fig. 1F), closely satisfy the condition for an ideal chiral point emitter where electric and magnetic dipoles have the equal contribution with a ±π/2 phase difference (see note S2). To quantify the chiral nature of our polaritons and draw an analogy with molecular enantiomers we evaluate the dissymmetry factor *g*, defined as the ratio of rotational strength *R* to dipole strength *D* (*46*)

$$g=\frac{4R}{D}=\frac{4\text{Im}\left(2M\cdot cP\right)}{{|2M|}^{2}+{|cP|}^{2}}$$ (1)

where **P** and **M** denote the electric and magnetic dipole moments, respectively. Since we consider polaritons confined within the bulk meta-atoms, whose sizes are much larger than those of typical molecular enantiomers, we cannot neglect the contribution of the electric quadrupole, which is of the same order in the multipole expansion as the magnetic dipole. This results in an extra doubling of **M** in Eq. 1 (see note S2 for more details). The numerically calculated values of *g* for two representative scaling factors on both polariton branches approach the theoretical upper limit of *g*_max_ = 2, confirming that the resulting polaritonic eigenstates very closely approach the condition of maximum optical chirality. The energy level diagram for this process in a left-handed metasurface is shown in Fig. 1G. This formation of self-hybridized chiral exciton-polaritons highlights our metasurface as a promising platform for chiral polaritonics.

### Maximally chiral metasurfaces for self-hybridized chiral exciton-polaritons

**Fig. 2.**
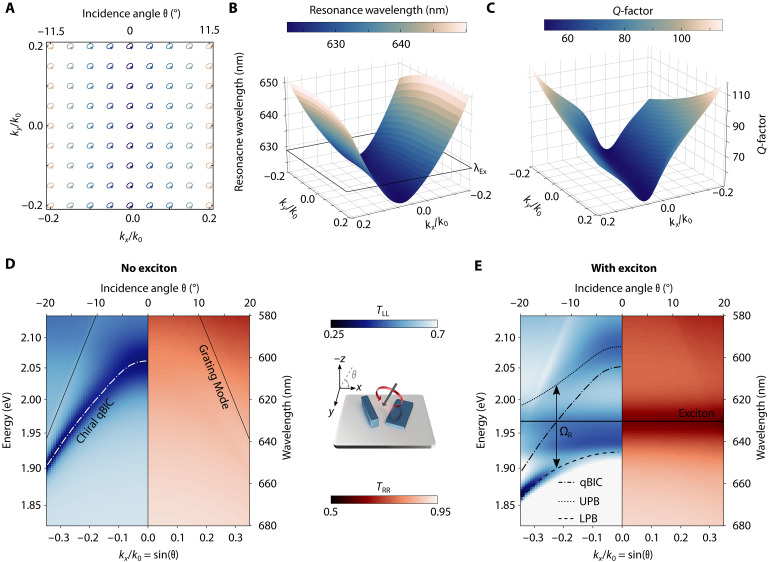
Angular dispersion analysis of chiral strong coupling. (**A**) The simulated far-field polarization of the left-handed metasurface confirms that the chiral qBIC’s maximum chirality remains unaffected by oblique incidence. (**B**) Simulated dispersion for the left-handed chiral qBIC resonance in *k*-space shows a saddle-shape. (**C**) *Q*-factor tuning behavior for varying incidence angle θ. The general saddle shape for *Q* factor shows an additional narrowing of the line width when *k_x_* and *k_y_* have opposite signs. (**D**) Simulated copolarized LCP (left) and RCP (right) transmittance spectra of the left-handed WS_2_ metasurface for different incidence angles θ in the *yz*-plane and without the exciton. The chiral qBIC sustains its polarization when moving to oblique incidence, enabling postfabrication tuning of the resonance wavelength via the incidence angle θ. (**E**) Simulated copolarized transmission of LCP (left) and RCP (right) light for different incidence angles θ of the metasurface with the exciton. LCP spectra show anticrossing formation while only the constant excitonic fingerprint is visible for RCP excitation.

Although the concept of qBICs in metasurfaces composed of nanorods with differing heights and antiparallel dipole configurations has been previously demonstrated (*42*, *43*), the *k*-space characteristics of such systems have remained unexplored. Nevertheless, there is an interest in momentum-space studies of chiral modes and their hybridization with excitonic materials (*47*). Here, we analyze the far-field polarization of the eigenstates supported by the metasurface structure depicted in Fig. 1 (with in-plane scaling factor *S* = 1 and, for more clarity, we neglect refractive index dispersion and set *n_xx_* = *n_yy_* = 4.5 and *n_zz_* = 2.55). As shown in Fig. 2A, the mode preserves its circular polarization throughout the entire *k*-space region under consideration, demonstrating improved robustness against variations in the angle of incidence compared to previously reported systems (*41*). Concurrently, the resonance wavelength of the chiral qBIC exhibits a saddle-shaped dispersion as a function of in-plane wave vector *k*, redshifting with increasing |*k_x_*| (Fig. 2B). This behavior enables continuous spectral tuning of the resonance position. Notably, the *Q* factor increases with |*k_x_*| but remains within experimentally feasible values, not exceeding 120 (Fig. 2C). Therefore, the angle of incidence no longer acts as a constraint on a system, but rather as a postfabrication degree of freedom for subnanometer precise resonance wavelength control and tuning. By varying the angle of incidence, the resonance wavelength of the chiral qBIC can be precisely tuned over a spectral range exceeding 50 nm. This allows for spectral shift across the excitonic resonance of WS_2_ at 629 nm (Fig. 2B), thus enabling to probe the chiral strong coupling using a single metasurface.

To illustrate this effect, we simulate the copolarized transmission spectra *T*_RR_ and *T*_LL_ as a function of *k_x_* (with *k_y_* = 0), using a metasurface geometry with *S* = 0.95 to shift the normal incidence qBIC resonance wavelength toward 600 nm. In the absence of the excitonic response (material model defined in Supplementary Note 1), the chiral qBIC mode appears exclusively in *T*_LL_ and shifts to longer wavelengths with increasing *k_x_* (Fig. 2D). Even at large in-plane momentum values (*k_x_* = 0.34, corresponding to an incidence angle θ = 20°), the mode maintains its chiral character. Incorporating the excitonic contribution into the permittivity model leads to the formation of two polaritonic branches, clearly resolved in the *T*_LL_ spectra (Fig. 2E), using only a single metasurface. In contrast, *T*_RR_ reveals no hybridized resonances and only a weak signature of the exciton. This behavior highlights the metasurface as a continuously tunable photonic platform, enabling noninvasive and reversible resonance control without the need for externally responsive materials, in contrast to conventional cavity systems (*40*).

### Experimental chiral strong coupling

**Fig. 3.**
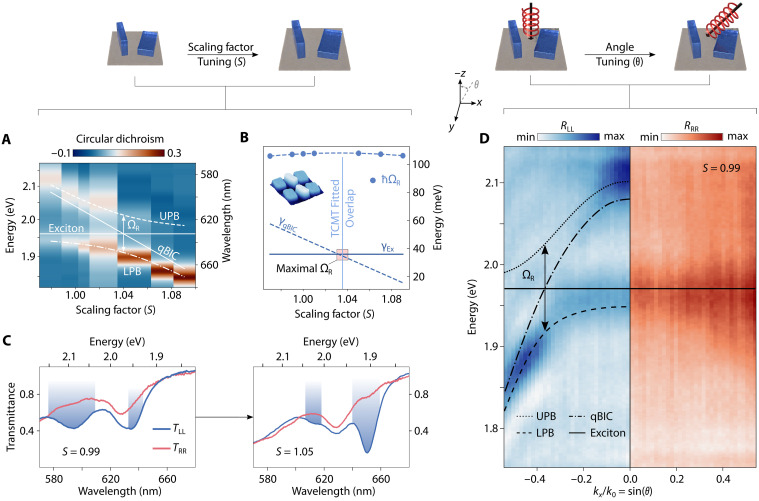
Experimental chiral strong coupling in parametric and *k*-space. (**A**) Tuning of the chiral qBIC resonance via scaling factor *S* results in polaritonic splitting into upper and lower branches. The dashed lines show the polariton dispersion extracted via TCMT fitting, yielding a Rabi splitting of 108.06 meV. (**B**) Extracted total linewidths for chiral qBIC $\gamma_{\text{qBIC}}$ and exciton $\gamma_{\text{Ex}}$ from TCMT fits, along with the fitted Rabi energy. At the overlap position, the fitted linewidths of exciton and chiral qBIC are close to becoming equal, indicating that the system approaches the maximal Rabi splitting. The inset shows an AFM image of the metasurface structure. (**C**) Transmittance spectra under LCP and RCP excitation for different scaling factor geometries at normal incidence. (**D**) Experimental back focal plane imaging of the copolarized reflectance spectra *R*_LL_ and *R*_RR_. Dashed lines highlight the polaritonic dispersion in left-handed signal (*S* = 0.99).

To demonstrate the formation of chiral self-hybridized exciton polaritons in our experiments, we fabricate a series of chiral WS_2_ metasurfaces with a range of in-plane scaling factors from *S* = 0.97 to *S* = 1.09. Analysis of SEM images gave the following dimensions for the structure (for *S* = 1): widths of 130 and 63 nm, heights of 80 and 30 nm, lengths of 210 nm, and a periodicity of 370 nm, which also were confirmed by postfabricational simulations (note S4). The corresponding circular dichroism CD = (*T*_RR_ − *T*_LL_)/(*T*_RR_ + *T*_LL_) spectra show a clear anticrossing behavior, a preliminary indicator of strong coupling (Fig. 3A).

We use a temporal coupled mode theory (TCMT) model to fit the transmittance spectra (see note S5), allowing us to rigorously assess the coupling strength. Unlike conventional polariton dispersion analysis, this method provides direct access to both the dispersion and linewidth of the underlying qBIC, offering a more complete and quantitative characterization of the coupled system. The linewidth of the WS_2_ exciton is *ℏ*γ*_Ex_* = 36 meV. This value and the excitonic energy at *ℏ*ω*_Ex_* = 1.971 eV are kept fixed for the TCMT fits used to extract the coupling strength. We further assume that the scaling factor and the qBIC dispersion follow a linear relationship (Fig. 3B), a common approximation in literature (*48*). The Rabi energy is defined as

$$\hbar \Omega_{R}=2\hbar \sqrt{\kappa^{2}-{\left(\frac{\gamma_{\text{qBIC}}-\gamma_{\text{Ex}}}{4}\right)}^{2}}$$ (2)

where Ω_R_ denotes the Rabi frequency and κ is the coupling strength. On the basis of our TCMT fitting, we obtain a Rabi splitting of *ℏ*Ω_R_ = 108 meV and κ = 13.1 THz.

To verify that the system operates in the strong coupling regime, it is essential to assess whether the rate of coherent energy exchange between the chiral qBIC and the exciton mode exceeds the rate at which each resonance dissipates energy into its respective loss channels. This condition ensures that the light-matter interaction gives rise to hybridized polariton modes with well-resolved, spectrally separable features. The first commonly used criterion *c*_1_ compares the Rabi frequency Ω_R_ with the sum of the linewidths of the exciton and qBIC modes (*49*)

$$c_{1}=\frac{\Omega_{R}}{\gamma_{\text{qBIC}}+\gamma_{\text{Ex}}}>1$$ (3)

indicating that the polariton mode splitting should exceed the total dissipation rate of the uncoupled modes. For our system, the calculated *c*_1_ = 1.5 clearly satisfies this criterion. A more stringent condition is given by the second criterion *c*_2_, which accounts for unequal linewidths and compares the coupling strength κ with the root mean square average of the losses (*49*)

$$c_{2}=\frac{\kappa }{\sqrt{\frac{\gamma_{\text{Ex}}^{2}+\gamma_{\text{qBIC}}^{2}}{2}}}>1$$ (4)

Our system also meets this stricter condition, with a calculated *c*_2_ = 1.5. Combining this with the observation of distinct anticrossing behavior, our metasurface is clearly within the chiral strong coupling regime.

The TCMT analysis further reveals that the system can be tuned to maximize Rabi splitting via straightforward geometrical variations. This is achieved by balanced linewidths of both exciton and chiral qBIC, which maximizes the observed spectral separation between the LPB and UPB (Fig. 3B). At normal incidence (*k_x_* = 0), different scaling factors exhibit the expected chiral response originating from the hybridization of maximally chiral qBIC and exciton: the left-handed signal *T*_LL_ shows pronounced UPB and LPB dips, whereas the right-handed trace *T*_RR_ shows only the bare excitonic feature (Fig. 3C). This further confirms that the chiral qBIC continues to couple selectively to LCP even as the exciton-photon detuning is varied via geometric scaling. We note that the far-field response is maximally chiral, with the chiral qBIC and polariton branches confined to *T*_LL_; however, residual interaction with RCP light remains (*T*_RR_ < 1), as evidenced by the excitonic resonance in *T*_RR_. Thus, a similar monolayer-integrated design could not realize full valley selectivity.

To probe the chiral character of the exciton-polariton modes within a particular metasurface, we leverage the previously established circular polarization stability in *k*-space of our design to perform angle-resolved reflectance measurements under circularly polarized illumination (see Materials and Methods), scanning the in-plane momentum *k_x_*/*k*_0_ = sin(θ), where θ is the angle of reflectance. Spectra were recorded separately for LCP and RCP light (*R*_LL_ and *R*_RR_) using back focal plane spectroscopy. The resulting dispersion in *R*_LL_ reveals clear signatures of strong coupling between the exciton and the chiral qBIC photonic mode, giving rise to UPB and LPB with a Rabi splitting that emerges at finite in-plane momentum (see note S6). In contrast, apart from a faint background that mirrors the bare excitonic absorption, no resonant chiral features are observed in *R*_RR_ (Fig. 3D).

Side-by-side comparison of the two dispersions reveals the momentum-resolved “fingerprint” of a metasurface whose optical response depends on the handedness of the incident light. The engineered chiral qBIC serves as a selective interface between photons and excitons, enabling efficient self-hybridization of chiral exciton-polaritons for LCP while remaining passive for RCP. We note that the chirality of our system is photonic in origin; the only linear-optical feature that requires polaritons is the helicity-selective anticrossing presented in Fig. 3. Moreover, we provide calculated Hopfield coefficients for scaling factor and in-plane-momentum sweeps, quantifying the excitonic-photonic composition of the resulting chiral polaritons (note S7).

### Chiral third-harmonic generation in the strong coupling regime

**Fig. 4.**
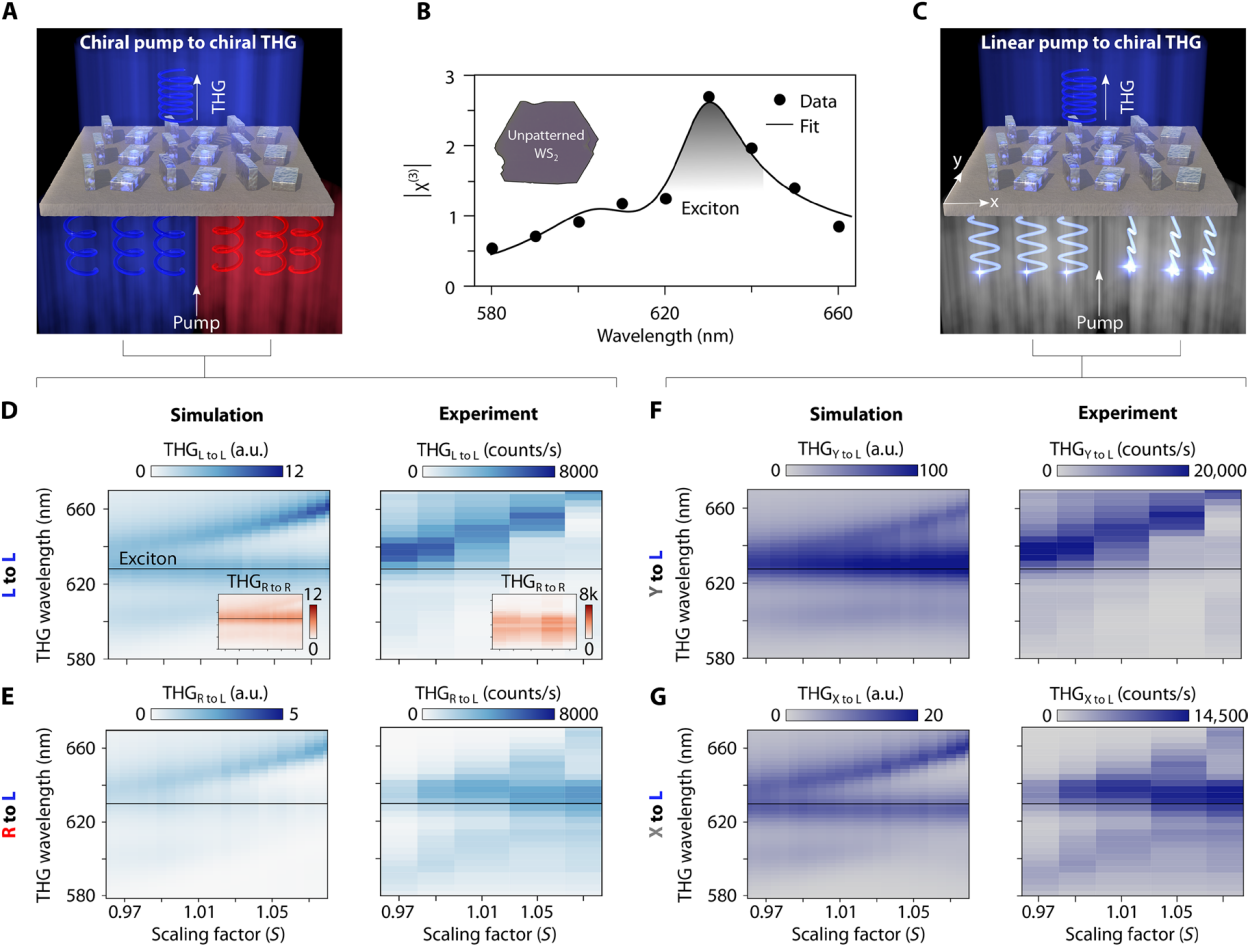
Chiral exciton-polaritons in THG. (**A**) Conceptual visualization of the chiral pumping harmonic generation experiment, where the emission is resonant with the chiral qBIC. (**B**) Absolute value of nonlinear susceptibility χ^(3)^ of unpatterned bulk WS_2_ extracted from experimental data, showing a native excitonic nonlinear enhancement. (**C**) Conceptual visualization of the linear pumping driven chiral harmonic generation experiment, again with emission resonant with the chiral qBIC. (**D**) Simulated and experimental left copolarized THG, exhibiting hybridization of the exciton and the chiral qBIC at 3ω. The insets show right copolarized THG only displaying native excitonic signal. (**E**) Right to left cross-polarized THG, again exhibiting exciton-qBIC hybridization in the emitted TH. The simulated and experimental left handed THG from linear pump, also exhibits hybridization of the exciton and the THG despite the linear pump polarization along (**F**) *Y* or (**G**) *X*.

In centrosymmetric bulk WS_2_ third-harmonic generation (THG) provides a nonlinear optical window into electronic and excitonic properties. When strong light-matter coupling is realized, the formation of exciton-polaritons modifies the optical response, including in the nonlinear regime. By resolving THG under circularly and linearly polarized excitation (Fig. 4, A and C), the polarization-dependent signatures of these hybrid states can be accessed.

Our nonlinear measurements on unpatterned WS_2_ flakes reveal a pronounced intrinsic enhancement of nonlinearities near the exciton resonance (Fig. 4B and note S9). Outside this excitonic region, the THG intensity decreases by more than an order of magnitude, corresponding to a reduction of the nonlinear susceptibility by over a factor of three. Note that this reflects a simplified nonlinear model, fitting only |χ^(3)^ (λ)| while neglecting off-diagonal tensor elements including their possible complex phases as well as various experiment-related factors.

In most qBIC-based nonlinear studies, the pump is tuned to the qBIC resonance so that the local field enhancement amplifies the nonlinear response of the material. Here, however, the pump is tuned between 1740 nm and 2000 nm in steps of 20 nm making the third-harmonic emission itself occur in the resonant wavelength range. This choice is dictated by the fundamental optical and electronic properties of the material: pumping at the exciton wavelength would shift the third harmonic into the ultraviolet, where WS_2_ is strongly absorbing. Comparing chiral THG intensities from metasurfaces with different scaling factors reveals that the handedness-dependent hybridization between the chiral qBIC and the exciton also governs the nonlinear response (Fig. 4D). Here, the WS_2_ exciton exhibits strong third-order nonlinearity and couples to the optical chiral qBIC, giving rise to hybrid chiral polaritons, with the qBIC providing a polarization-selective radiative channel for the third-harmonic emission.

To further explore the origin of this nonlinear chirality, we must consider the field distribution within the metasurface, which results from the hybridization of the chiral qBIC mode with the exciton. When the left-handed metasurface is pumped by RCP light the generated nonlinear emission is governed not by the polarization state of the incident light, but by the intrinsic handedness of the chiral polariton. Consequently, even RCP excitation yields a LCP third-harmonic signal (Fig. 4E). Only the excitonic response can be observed in the corresponding RCP third-harmonic signal (Fig. 4D). We further show (note S13) that, in the weak coupling regime, the qBIC provides a chiral radiative channel and gives a finite chiral dissymmetry to the exciton THG emission.

To show that the effect is not limited to circularly polarized pumping, we measured THG under linearly *X*- and *Y*-polarized excitation (Fig. 4, F and G). Simulations and experiments alike reveal a pronounced polariton-induced splitting in the LCP third-harmonic signal, while the RCP signal remains weak. This indicates that the branch-resolved nonlinear spectral features originate from exciton-qBIC hybridization, whereas the underlying chiral bias is imposed by the chiral qBIC radiative channel rather than by the pump polarization or intrinsic material chirality. A full comparison of simulated and measured THG under different pump polarizations, as well as the experimental THG efficiency, is provided in notes S11 and S12, respectively.

Transforming a purely linear pump into helicity-controlled harmonic emission within a passive, complementary metal-oxide semiconductor–compatible dielectric metasurface opens several avenues. This control arises not from the light source or material chirality, but from the geometry-defined chiral qBIC, which in the strong-coupling regime gives rise to chiral exciton-polaritons. The metasurface design, parameterized by an opening angle α, enables compact, ultrafast sources of circularly polarized light (CPL) for chiral spectroscopy and valley-exciton oriented 2D material control. Left- and right-handed elements can be integrated for helicity-multiplexed channels without active optics. In addition, polariton-induced spectral splitting offers tunable frequency control for nonlinear signal processing. This approach connects chiral photonics with polariton physics for on-chip, helicity-engineered light sources.

## DISCUSSION

We have developed an out-of-plane symmetry-broken metasurface fabricated from bulk TMDC to demonstrate and experimentally verify chiral self-hybridized exciton-polaritons, pushing beyond two major bottlenecks of cavity-polariton physics. First, the generation of chiral fields is unattainable in conventional closed Fabry-Pérot cavity systems because of phase-flipping at mirror interfaces (*50*). The need for a handedness-preserving mirror has already been understood and experimental proof-of-concept metamaterial designs were proposed in the gigahertz range (*51*) as well as theoretically investigated in the visible range (*24*). For the latter theoretical study, handedness-preservation was achieved by hybridization of different parity eigenstates. However, experimental realizations of more general chiral polaritonic cavity approaches have remained elusive.

By introducing the physics of metasurfaces with maximum chiral qBICs, we elevate the concept of chiral cavities, realizing a platform that preserves the handedness of a localized high-*Q* optical mode in the visible range, while offering exceptional design flexibility and dynamic tunability. As a result, we observe the formation of chiral self-hybridized exciton-polaritons. Second, the metasurface resonance is tunable over a substantial range by using the angle of incidence as noninvasive postfabrication tuning mechanism without adversely affecting the chiral performance. This presents a step forward for polaritonic physics, as it provides a clear pathway towards not only chiral cavities but also greatly simplified detuning without the need for highly specialized stimuli-responsive materials (*52*) or complex electrically driven designs (*53*).

By leveraging the out-of-plane symmetry breaking of our chiral qBIC metasurface, we reveal chiral polaritonic signatures in the third-harmonic emission of a bulk TMDC. The nonlinear process is radiatively mediated by the chiral qBIC and hybridizes with the A-exciton, yielding a spectrally split, circularly polarized THG output, even under a linearly polarized pump. This control over bulk nonlinearities via exciton-polariton opens avenues for chip-scale quantum light emitters (*54*), ultrafast spin-selective light sources (*55*), and reconfigurable nonlinear mirrors (*56*). Because the platform uses bulk vdW materials and high-quality centimeter-scale TMDC films can now be routinely obtained by vapor deposition (*57*), the architecture is highly scalable. In particular, its ability to translate widely available linearly polarized laser inputs into helicity-programmed, frequency-converted outputs makes it an attractive candidate for integrated optical parametric generation and amplification in advanced photonic circuits (*58*). Through our generally applicable chiral metasurface design, the same strategy can be extended to material systems that exhibit strong intrinsic polaritonic or nonlinear responses, including halide perovskites, magnetic vdW crystals such as CrSBr, and Moiré-twisted heterostructures. Implementing chirality-driven symmetry breaking in these emerging quantum materials could enable tailored polariton landscapes, valley-specific nonlinearities and other symmetry-protected phenomena, paving the way toward a class of quantum-engineered photonic devices.

## MATERIALS AND METHODS

### Numerical simulations

The refractive index of the SiO_2_ substrate was set as 1.45, while that of the WS_2_ rods was taken from literature (*59*). Simulations of transmittance spectra for the 3D-chiral WS_2_ metasurfaces were conducted using CST Studio Suite 2021 with periodic Floquet boundary conditions. Far-field polarization and transmittance behavior under oblique incidence were numerically investigated using the Electromagnetic Waves Frequency Domain module of COMSOL Multiphysics in 3D mode using a previously developed approach (*60*). The tetrahedral spatial mesh for FEM was automatically generated by COMSOL’s physics-controlled preset. Simulations were performed within a rectangular spatial domain containing a single metasurface unit cell with periodic boundary conditions applied to its sides. Circularly polarized ports were set at the top and bottom to simulate excitation and registration. To simulate THG two problems at fundamental and third-harmonic wavelengths were solved simultaneously using COMSOL Multiphysics. To connect two studies a polarization node was set up with the fields of the linear problem in the volume of the rods.

### Sample fabrication

Fused silica substrates were initially cleaned by sonication in acetone at 55°C, followed by isopropanol to remove any residual acetone. Subsequently, the substrates were treated with O_2_ plasma to eliminate organic residue and enhance flake adhesion. To facilitate precise global alignment of the flake position on the substrate during subsequent processing, a marker system was created on the substrates using optical lithography (SÜSS Maskaligner MA6). WS_2_ flakes were mechanically exfoliated from bulk crystals (HQ Graphene) onto the cleaned silica marker substrates. The deposition process was conducted at a temperature of 105°C to evaporate moisture and stretch the exfoliation tape, ensuring flattened transferred flakes. The height of the flakes was measured using a profilometer (Bruker Dektak XT) with a stylus having a radius of 2 μm. The three-dimensional WS_2_ metasurfaces were fabricated using a multistep E-beam lithography (EBL) process, followed by lift-off and reactive ion etching (RIE). All EBL steps were carried out using an eLINE Plus (Raith Nanofabrication). For the first EBL step, the films were spin-coated with a positive electron beam resist, CSAR 62 (Allresist) and Espacer 300Z. The right half of the unit cell was fully exposed via electron beam lithography using an eLINE Plus system (Raith) at 30 kV with a 15-μm aperture. The patterns were developed in an amyl acetate bath, followed by a MIBK:IPA (1:9 ratio) bath. The intended height difference was etched into the flakes via RIE (Oxford PlasmaPro 100) using SF_6_-based chemistry at a pressure of 20 mTorr and a radio-frequency power of 50 W using the unexposed resist as a hardmask. Subsequently, a single layer of positive-tone polymethylmethacrylate (Kayaku Advanced Materials) was used as the EBL resist, spin-coated at 3000 RPM and baked at 180°C for 3 min. Espacer 300Z was then spin-coated onto the sample. The full unit cell design was patterned with the same EBL system at 20 kV with a 15-μm aperture. Development took place in a solution of 40-ml ethanol mixed with 7.5 ml of deionized water for 20 s. Before the first EBL step, a local marker system (50-nm-thick Au) was installed in direct proximity to the flake for precise realignment during subsequent steps. After the second patterning run, a hardmask consisting of 2.5-nm titanium, followed by 40 nm of gold evaporated onto the sample using electron beam evaporation and subsequently lifted off overnight in Microposit Remover 1165. The remaining flake was then etched through via RIE, and the Au hardmask was removed using a solution of potassium monoiodide and iodine (Sigma-Aldrich).

### Chiral optical characterization

The chiral optical characterization was conducted using a custom-built transmission microscope (sketch see Supplementary Text). The system was driven by a fiber-coupled supercontinuum white light laser (SuperK FIANIUM from NKT Photonics) set to 5 to 8% of its maximum power and a repetition rate of 0.7 to 1.8 MHz. The laser beam was directed through a polarizing beam splitter, dividing it into horizontal (HP) and vertical (VP) linearly polarized components (2x LPVIS100 from Thorlabs, 550 to 1500 nm). A quarter-wave plate (QWP; RAC4.4.20 from B-Halle, 500 to 900 nm) was used to generate CPL. By blocking the HP or VP path, the polarization could be adjusted between RCP and LCP. This method avoided the need to rotate polarizers or the QWP, which can introduce elliptical polarization if not carefully controlled. The QWP was positioned directly below the objectives to prevent reflections from mirrors, which can convert CPL into elliptically polarized light. The light was condensed onto the sample using a 10× objective [Olympus PLN, numerical aperture (NA) = 0.25] for normal incidence scaling factor measurements and a 20× objective (Olympus PLN, NA = 0.40) for incidence angle measurements. The light was collected using a 60× objective (Nikon MRH08630, NA = 0.7). The beam was condensed to illuminate the entire metasurface area of 30 μm by 30 μm. The incidence angle measurements were performed by rotating the sample holder. For the measurement of co- and cross-polarization terms, a chiral analyzer consisting of a QWP (AQWP05-580 from Thorlabs, 350 to 850 nm) and a linear polarizer (WP25M-UB from Thorlabs, 250 to 4000 nm) were installed after the collection objective. A flip mirror was used to direct the light either directly to a charge-coupled device (CCD) camera or to a spectrometer via a multimode fiber (Thorlabs M15L05, core size: 105 μm, NA = 0.22). A spectrometer from Princeton Instruments with a grating period of 300 g/mm, blazed for 750 nm, and a spectral resolution of 0.13 nm was used. All spectra were recorded with a binning of six lines, an exposure duration of 90 ms, and 20 accumulated spectra. All spectra were normalized using a background measurement taken on the same substrate using the matching angle of incidence and co-/cross-polarization. To verify the handedness and degree of circular polarization at the sample plane, we used a calibrated polarimeter (Polarization Analyzer SK010PA-UVIS, Schäfter+Kirchhoff; 400 to 700 nm). The measured phase shift lies between 43° and 45° for both left- and right-circularly polarized paths, corresponding to near-ideal circular polarization (45°); the 43° lower bound implies a ≈4.5% deviation, consistent with the manufacturer-specified retardance tolerance (~6%). The measured degree of circular polarization is ≈100% above 550 nm, i.e., across the spectral range relevant to this work.

### *k*-space optical measurements

The *k*-space angle-resolved measurements were obtained using a homebuilt optical setup that operates in back-reflection geometry (see Supplementary Text). A halogen lamp (Thorlabs, SLS201L) served as the excitation light source. The collimated light was directed through a 50:50 beamsplitter to the 60× magnification objective (NA = 0.95) and the sample. The same objective was used to collect the reflected light. A Fourier lens was used to image the back focal plane of the objective. With the assistance of a second lens, we created a 4-f system that projected the back focal plane image onto the slit of the spectrometer. To control the polarization of the light, two linear polarizers (LPVISC100-MP2) and one quarter-wave plate (Thorlabs λ/4, 400 to 800 nm) were used. As the light passed through the first linear polarizer and the quarter-wave plate rotated at 45°, it became circularly polarized. The reflected light then traversed the quarter-wave plate again, converting it back to linearly polarized light. The orientation of the second linear polarizer acted as an analyzer, enabling the performance of angle-resolved measurements with circular polarization information. The image projected onto the spectrometer slit (Princeton Instruments) will be expanded according to the wavelength, aided by a spectrometer grating (150 g/mm, blazed for 800 nm). The resulting image is then projected on the CCD sensor coupled at one exit port of the spectrometer.

### Nonlinear optics experiments

Harmonic generation experiments used a 140 fs, 80 MHz mode-locked Ti:sapphire laser (Chameleon Ultra II) pumping an optical parametric oscillator (Chameleon compact OPO) for generating tunable infrared pulses using its idler output (see Supplementary Text). The beam passed a Glan-Taylor prism and a broadband infrared half-wave plate (HWP) for linear polarization control, as well as a QWP for experiments with circular polarized excitation. A 10×, 0.25-NA objective focused the beam on the sample, while another 10×, 0.25-NA objective collected the third harmonic. A broadband visible QWP was then used for converting circular polarized emission into linear polarized light, and a following HWP and linear polarizer were used to control the collection polarization or handedness. The signal was detected with a spectrometer (Acton SP2300) using a silicon CCD camera (Pixis 100F). For a sketch see Supplementary Text. Polarization at focus was not recharacterized; however, substantial degradation of circular polarization at the focus is unlikely, as no mirrors or other polarization-altering optics are present between the QWP and the sample, the linear input is defined by a polarizer upstream of the QWP, and the waveplate retardance remains within 0.246 to 0.253 across the operating band.

## Supplementary Materials

### This PDF file includes:

Supplementary Notes S1 to S14 Figs. S1 to S17 References
