# Supplementary material for: Chiral nonlinear polaritonics with van der Waals metasurfaces
Source: Sci Adv. 2026 Mar 27;12(13):eaeb5631. doi: 10.1126/sciadv.aeb5631 (PMC13025102; doi:10.1126/sciadv.aeb5631)
Supplement: Supplementary file 1 — Supplementary Notes S1 to S14 Figs. S1 to S17 References [file sciadv.aeb5631_sm.pdf]

Science Advances
AAAS

Supplementary Materials for

Chiral nonlinear polaritonics with van der Waals metasurfaces

Connor Heimig *et al.*

Corresponding author: Andreas Tittl, andreas.tittl@physik.uni-muenchen.de

Sci. Adv. **12**, eaeb5631 (2026)
DOI: 10.1126/sciadv.aeb5631

This PDF file includes:

Supplementary Notes S1 to S14
Figs. S1 to S17
References

Supplementary Note 1: Unit Cell Design Parameters

While the period, resonator dimensions, and positioning within the unit cell are all significant parameters for qBIC mode formation, the two pivotal design parameters for achieving maximum chirality are illustrated below in Figure S1. These are the height difference, Δh, and the opening angle, which should be in-line with the theoretical relation $\alpha \sim k\Delta h$ for the maximal chiral response (*42*). The height difference is the core parameter for 3D chirality and is established during fabrication. The influence of increasing Δh on maximum chirality is initially pronounced before eventually reaching a plateau (Fig. S1a). This indicates that the necessity for ultra-precise etching of the height difference with nanometer precision is reduced, provided that a benchmark Δh is surpassed. Therefore, a tendency towards overetching proved an advantageous approach, as it has only very limited downside for the overall mode formation and chirality of the qBIC resonance, in strong contrast to underetching, resulting in too small Δh.

The sign of the opening angle α regulates the system's preferred handedness, while simultaneously modulating the chirality and linewidth of the resonance (Fig. S1c). To resolve the upper polariton branch at wavelengths below the exciton, an opening angle $\alpha = 12°$ was deemed optimal for the experiment. This angle was selected to broaden the resonance linewidth, thereby ensuring the qBIC mode would be robust against the higher intrinsic material losses of WS_2 in this spectral region (Fig. S1b). All simulations and experiments were conducted using positive opening angles, resulting in left-handed structures throughout this work for the sake of clarity.

This allows the design to be optimized in such a way that the qBIC resonance is maximally chiral and has spectral overlap with the A-exciton in bulk WS_2 at room temperature (Fig. S1d). Upon switching to the material model including the exciton, the formation of qBIC-driven chiral self-hybridized exciton-polaritons becomes apparent (Fig. S1e). Accordingly, the emergence of upper and lower polariton branches (UPB, LPB) in left-handed transmittance is observed, whereas the right-handed signal only exhibits the excitonic peak. To illustrate the behavior of our maximally chiral qBIC for both the non-excitonic and the excitonic WS_2, we introduce the transmission difference $\Delta T = T_{RR} - T_{LL}$ and examine the spectral dispersion of the pure qBIC signal when shifting the resonance via in-plane scaling factor S of the unit cell (Fig. S1f). In the case of the non-excitonic material, the qBIC exhibits a linear trend. In contrast, when moving to the excitonic WS_2 material model, an anticrossing pattern emerges, which is characteristic for strongly coupled systems. Furthermore, since the transmission difference is studied, it is evident that this strong coupling only occurs in the left-handed signal, thereby proving the formation of self-hybridized chiral polaritons in the system.

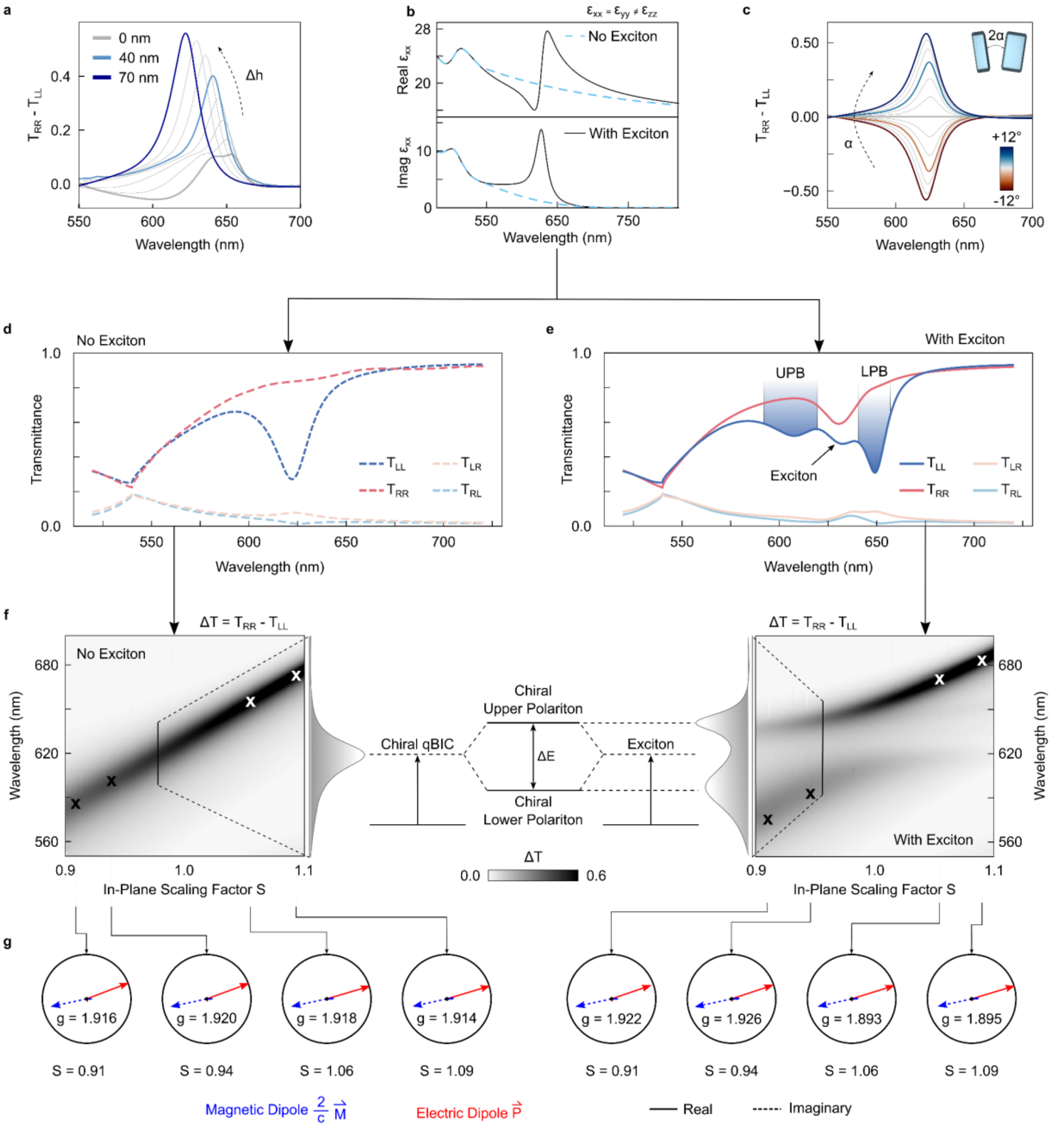

Fig. S1. Unit cell parameters and material model.
Dependence of T_{RR} - T_{LL} on **(A)** height difference and **(C)** opening angle α. **(B)** Material Data of WS_2 was taken from Mukhbat et al. (*59*) and adapted to model the material dispersion both with and without the excitonic contribution. **(D)** Chiral transmittance spectra (LCP/RCP) of the left-handed metasurface without the influence of the exciton for both co- and cross-polarization, highlighting the maximum chirality of the qBIC response. **(E)** When including the exciton, the chiral transmittance spectra show the emergence of self-hybridized exciton-polaritons. **(F)** Simulated transmittance differences (T_{RR} - T_{LL}) of the chiral WS_2 qBIC metasurfaces for different in-plane scaling factors S using the material models with and without the exciton. The simulations with the exciton show a characteristic anticrossing pattern around the exciton. The metasurfaces dimensions are listed in the caption for Fig.1E of the Main Text. **(G)** Multipole decomposition of eigenstates with contributions of electric and magnetic dipole moments having

almost the same amplitude and phase difference close to π/2. g is the dissymmetry factor, defined according to Eq. 1 from the main text.

Supplementary Note 2: Multipole Decomposition for Chiral Polaritons

The coupling coefficient of an eigenstate of a metasurface with a normally incident plane wave (along the z-direction) with a wave number $k = \omega/c$ and polarization unit vector **e** can be evaluated as an overlap integral:

$$m_e \propto \int_V \mathbf{J}(\mathbf{r}) \cdot \mathbf{e}e^{i\mathbf{k}\cdot\mathbf{r}}dV \approx \int_V \mathbf{J}(\mathbf{r}) \cdot \mathbf{e}(1 + i\mathbf{k}\cdot\mathbf{r})dV$$

$$\approx -i\omega\mathbf{P}\cdot\mathbf{e} - i(\mathbf{k}\times\mathbf{M})\cdot\mathbf{e} + \frac{\omega}{6}(e_\alpha)(k_\beta)Q_{\alpha\beta} \quad \text{(S1)}$$

where $\mathbf{J}(\mathbf{r})$ is an eigenstate displacement current density of the metasurface. The electric and magnetic dipole as well as the electric quadrupole moments are introduced in the standard way (*61*):

$$\mathbf{P} = \frac{i}{\omega}\int_V \mathbf{J}(\mathbf{r})dV \quad \text{(S2)}$$

$$\mathbf{M} = \frac{1}{2}\int_V \mathbf{r}\times\mathbf{J}(\mathbf{r})dV \quad \text{(S3)}$$

$$Q_{\alpha\beta} = \frac{3i}{\omega}\int_V \left[r_\alpha J_\beta(\mathbf{r}) + r_\beta J_\alpha(\mathbf{r}) - \frac{2}{3}\delta_{\alpha\beta}\mathbf{r}\cdot\mathbf{J}(\mathbf{r})\right]dV \quad \text{(S4)}$$

We consider circularly polarized incident waves with $\mathbf{e}_\pm = \frac{1}{\sqrt{2}}(\mathbf{e}_x \pm i\mathbf{e}_y)$ and reduce Eq. (S1) to the following:

$$m_\pm \propto [\mathbf{P}_x \pm i\mathbf{P}_y] \pm \frac{i}{c}[\mathbf{M}_x \pm i\mathbf{M}_y] + \frac{i\omega}{6c}[Q_{xz} \pm iQ_{yz}] \quad \text{(S5)}$$

If the eigenstate current $\mathbf{J}(\mathbf{r})$ flows predominantly in the xy-plane ($J_z \approx 0$), the components of the moments in Eqs. (S2–S4) contributing to Eq. (S5) then approximately reduce to:

$$\mathbf{P}_x = \frac{i}{\omega}\int_V J_x(\mathbf{r})dV, \quad \mathbf{P}_y = \frac{i}{\omega}\int_V J_y(\mathbf{r})dV \quad \text{(S6)}$$

$$\mathbf{M}_x = -\frac{1}{2}\int_V zJ_y(\mathbf{r})dV, \quad \mathbf{M}_y = \frac{1}{2}\int_V zJ_x(\mathbf{r})dV \quad \text{(S7)}$$

$$Q_{xz} = \frac{3i}{\omega}\int_V zJ_x(\mathbf{r})dV, \quad Q_{yz} = \frac{3i}{\omega}\int_V zJ_y(\mathbf{r})dV \quad \text{(S8)}$$

which leads to the following form of the coupling coefficient:

$$m_\pm \propto [\mathbf{P}_x \pm i\mathbf{P}_y] \pm \frac{2i}{c}[\mathbf{M}_x \pm i\mathbf{M}_y] \quad \text{(S9)}$$

Conventionally, chiral molecular electromagnetic scattering *(46)* and emission *(18)* are attributed to combinations of parallel electric **P** and magnetic **M** point dipoles with a ±π/2 phase difference $\mathbf{M} = \pm ic\mathbf{P}$ under the assumption of free molecular rotation implying averaging over its orientations. Conversely, here, as the metasurface orientation is fixed, the nonvanishing contribution from electric quadrupole effectively doubles the contribution from **M** and transforms the maximum chirality condition $m_\pm = 0$ into:

$$\mathbf{M} = \pm\frac{ic}{2}\mathbf{P} \quad \text{(S10)}$$

We employ the Eigenstate Solver of COMSOL Multiphysics to analyze the polariton fields. We set a constant WS_2 permittivity according to its tabular value at a fixed wavelength corresponding to a resonance position in Fig. S1f (with and without excitons, marked as crosses). By calculating the integrals in Eq. (S6) and Eq. (7S) we analyze the electric dipole moment in the form:

$$\mathbf{P} = \mathbf{P}' + i\mathbf{P}'' \quad \text{(S11)}$$

where the components of $\mathbf{P}'$ and $\mathbf{P}''$ are purely real. Since the eigenstate is found with an unknown phase ϕ, we define it as:

$$\tan(2\phi) = \frac{2\mathbf{P}'\cdot\mathbf{P}''}{(\mathbf{P}'')^2-(\mathbf{P}')^2} \tag{S12}$$

so that $\mathrm{Re}(\mathbf{P}e^{i\phi})\cdot\mathrm{Im}(\mathbf{P}e^{i\phi}) = 0$. Then we plot the real and imaginary parts of $\mathbf{P}e^{i\phi}$ in Fig. S1g and reveal, that the imaginary part of the electric dipole is negligibly small and hardly noticeable. see Fig. S1g). Next we analyze the magnetic dipole:

$$\mathbf{M} = \mathbf{e}_x M_x + \mathbf{e}_y M_y \tag{S13}$$

which depends on a coordinate system. Shifting the coordinate origin by $\mathbf{r}' = \mathbf{r}_0 + \mathbf{r}$ where $\mathbf{r}_0 = (x_0, y_0, z_0)^T$ leaves **P** unaffected, but changes **M**. Nevertheless, since the expansion in Eq. (S1) was performed in the vicinity of z = 0, we can slightly shift the coordinate origin by $\mathbf{r}_0 = (0,0,z_0)^T$ to match the center of the lower rod (by setting z_0 = 20 nm). The final magnetic dipole components read as:

$$M_x = -\frac{e^{i\phi}}{2}\left[\int_V z J_y(\mathbf{r})dV - z_0\int_V J_y(\mathbf{r})dV\right] \tag{S14}$$

$$M_y = \frac{e^{i\phi}}{2}\left[\int_V z J_x(\mathbf{r})dV - z_0\int_V J_x(\mathbf{r})dV\right] \tag{S15}$$

Finally, we plot the real and imaginary parts of $2c^{-1}\mathbf{M}e^{i\phi}$ (Fig. S1g) and show that and$2c^{-1}\mathbf{M}e^{i\phi}$are collinear and have similar amplitudes. It also becomes evident that the electric dipole contributes primarily through its real part, while the magnetic dipole contributes via its imaginary part, providing the phase difference of $\pi/2$ necessary for a chiral point emitter. Therefore, the above results indicate that $m_- \approx 0$ and the eigenstate is uncoupled from RCP light.

Supplementary Note 3: Three-Dimensional Metasurface Fabrication

In order to achieve a structure featuring resonators with different heights within each metasurface unit cell, a considerable fabrication process is necessary. To this end, WS_2 flakes were exfoliated onto fused silica substrates with flake thicknesses ranging from 70 nm to 115 nm. The sample was realigned between the patterning steps done via EBL and RIE (Methods) by using specific gold alignment markers. To realize the out-of-plane asymmetric metasurfaces presented in this work a two-step fabrication strategy is required that ensures precise alignment between resonators of different heights within a single unit cell. The presented method overcomes these limitations by first introducing a height reference structure directly into the 2D flake (Fig. S2a). In Step 1, a barcode-like pattern corresponding to exactly half of the metasurface unit cell is defined via electron beam lithography (1), developed (2), and the inverse of it transferred into the WS_2 flake using reactive ion etching (3). This produces a controlled step height (Δh) within the flake. As shown in (Fig. S2b), SEM images confirm the lateral pattern fidelity and etch depth, while profilometry verifies the uniformity and reproducibility of Δh. Larger etched rectangles within the pattern serve as references for measuring the height step. This inverse patterning methodology builds upon previous work. (*62*)
In Step 2 (Fig. S2c), the full metasurface pattern is defined in a single lithography step (4), overlaid onto the pre-etched barcode such that the high and low resonators fall on the corresponding height regions. In previous approaches, even minimal misalignment between lithography steps could significantly degrade pattern fidelity due to the strict spatial correlation required. (*43*) In contrast, our design intrinsically tolerates a much broader alignment window:

because the barcode spans exactly half the unit cell along x, the full metasurface resonators can be defined without high-precision alignment, while now achieving the highest possible fidelity. Furthermore, since all resonators are written within a single EBL step, perfect vertical and lateral registration between high and low segments is ensured. The only remaining alignment requirement is a lateral (x-axis) overlay of the metasurface pattern onto the barcode. Since the resonators do not span half a period L along x (their widths are approximately $\frac{L_x}{4}$ and $\frac{L_x}{3}$ for the slim and wide rods, respectively, and they are rotated by only 12°), any alignment within ±30 nm along the x-axis, which is well within standard EBL tolerances, yields perfectly accurate 3D chiral metasurface structures. Moreover, because the height differentiation barcode is continuously integrated into the flake along the y-axis, ideal alignment in this direction is intrinsically ensured.

Subsequent processing involves gold deposition and lift-off (5), followed by RIE (6) and wet etching (7) to transfer the pattern into the flake and remove the residual mask. As a result, this technique guarantees perfect intra-unit cell as well as global alignment between resonators and, compared to previous work, (*43*) offers a simplified process with substantially improved structural fidelity and reproducibility. The strategy effectively eliminates resonator misalignment and enables highly reproducible top-down 3D nanophotonic architectures. Furthermore, since the technique relies exclusively on etching, it is generally applicable to any resonator material system, including exfoliated, chemically grown, or vapor-deposited materials.

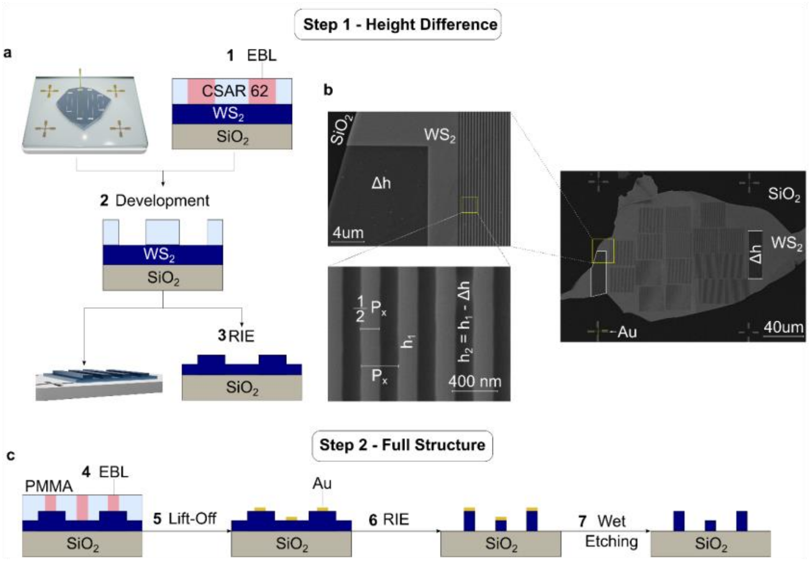

Fig. S2. Experimental realization of 3D chiral WS_2 metasurfaces.
(A) Outline of first inverse EBL step to establish height difference. **(B)** SEM images of WS_2 flakes after first step. The larger rectangles of etched material are used to determine the height difference via profilometer or AFM. **(C)** Outline of second fabrication step, allowing for simultaneous patterning of both high and low resonator, reducing the needed alignment accuracy to achieve maximum structural fidelity.

Supplementary Note 4: Comparison of Simulation and Experiment

To verify the experimental results from the corresponding section of the main text we compare them with numerical simulations, displaying an excellent agreement of theory and experiment.

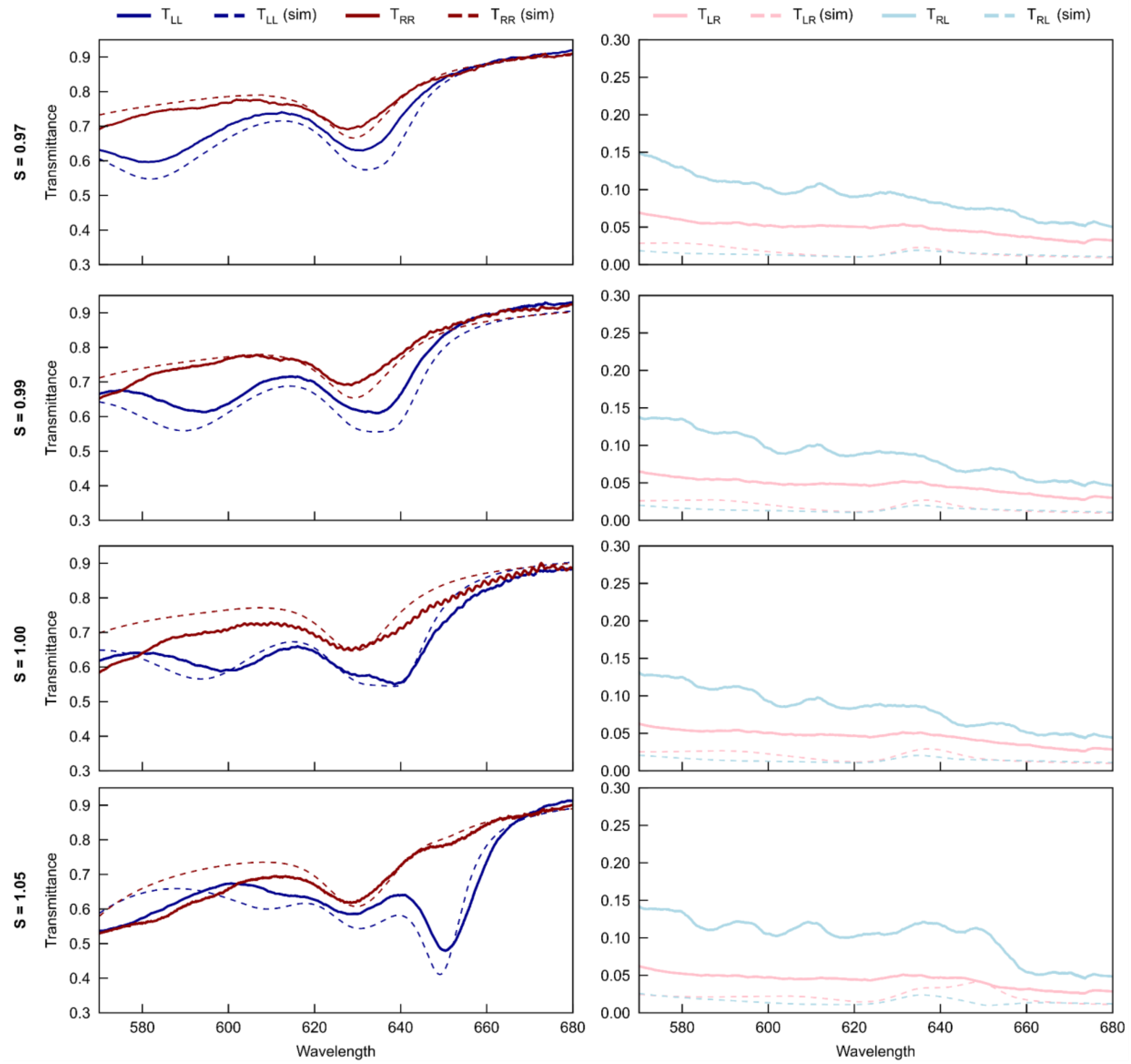

Fig. S3. Chiral transmission under normal incidence.

The unit cell design (for scaling factor $S = 1.00$) has a periodicity of $P_x = P_y = 370$ nm, two rods with the same length (210 nm) but different widths (130 nm and 63 nm) and heights (30 nm and 80 nm). Each rod is rotated by an angle of $\alpha = 12^{\circ}$. This design was used for the simulations in Fig. 4 in the main text.

Supplementary Note 5: Temporal Coupled-Mode Theory for qBIC–Exciton Coupling

The following section is based on the work of Fan et al. (*63*) on temporal coupled-mode theory (TCMT) for the Fano resonance in optical resonators. We consider a resonant system coupled to two ports, allowing for both transmission and reflection. The system is excited through port 1, with the incoming wave represented as

$$\boldsymbol{s}_+ = \begin{pmatrix} s_{1+} \\ 0 \end{pmatrix}, \tag{S16}$$

and the output waves in port 1 (reflected wave) and port 2 (transmitted wave) represented by

$$\boldsymbol{s}_- = \begin{pmatrix} s_{1-} \\ s_{2-} \end{pmatrix} \tag{S17}$$

where s_{1+}, s_{1-} and s_{2-} correspond to the incoming, reflected, and transmitted waves, respectively. In general, the scattering process is described by the scattering matrix *S* as

$$S(\omega) = C + K[i(\omega I - \Omega) + \Gamma]^{-1}K^T, \tag{S18}$$

where *C* describes the non-resonant port crosstalk, given by

$$C = e^{i\phi}\begin{pmatrix} r_0 & it_0 \\ it_0 & r_0 \end{pmatrix}, \tag{S19}$$

with r_0 and t_0 being the background reflection and transmission coefficients, respectively, constrained by $r_0^2 + t_0^2 = 1$. The phase ϕ is a global phase factor. The matrix Ω contains the complex resonance frequencies $\omega_j = \omega_{0,j} + i\gamma_{\text{int},j}$ on the diagonal, and the near-field coupling rates between the individual modes on the off-diagonal. The matrix Γ contains the radiative damping rates and extrinsic mode-coupling rates, defined as $\Gamma_{ij} = \sqrt{\gamma_{\text{rad},i}}\sqrt{\gamma_{\text{rad},j}}$ for $i, j = 1, \ldots, m$, where *m* is the number of resonant modes. The port-mode coupling is described by the matrix *K* with elements $K_{nj} = \sqrt{\gamma_{\text{rad},j}}$ for $n = 1, 2$.
The transmission spectrum is obtained from the scattering matrix as

$$T(\omega) = |S_{21}(\omega)|^2, \tag{S20}$$

and is used to fit experimental spectra. In our system, as described in (*44*), the matrices are given by

$$\Omega = \begin{pmatrix} \omega_{\text{qBIC}} + i\gamma_{\text{qBIC,int}} & \kappa & 0 \\ \kappa & \omega_{\text{Ex}} + i\gamma_{\text{Ex,int}} & 0 \\ 0 & 0 & \omega_{\text{Ex}} + i\gamma_{\text{Ex,int}} \end{pmatrix}, \tag{S21}$$

$$\Gamma = \begin{pmatrix} \gamma_{\text{qBIC,rad}} & 0 & \sqrt{\gamma_{\text{qBIC,rad}}\gamma_{\text{Ex,rad}}} \\ 0 & 0 & 0 \\ \sqrt{\gamma_{\text{qBIC,rad}}\gamma_{\text{Ex,rad}}} & 0 & \gamma_{\text{Ex,rad}} \end{pmatrix}, \tag{S22}$$

$$K = \begin{pmatrix} \sqrt{\gamma_{\mathrm{qBIC,rad}}} & 0 & \sqrt{\gamma_{\mathrm{Ex,rad}}} \\ \sqrt{\gamma_{\mathrm{qBIC,rad}}} & 0 & \sqrt{\gamma_{\mathrm{Ex,rad}}} \end{pmatrix}, \tag{S23}$$

$$C = e^{i\phi} \begin{pmatrix} r_0 & it_0 \\ it_0 & r_0 \end{pmatrix}, \tag{S24}$$

where κ denotes the coupling strength between the qBIC and the exciton.

To accurately fit the coupling strength, we employ an analysis adapted from Nan et al. (*48*), where we simultaneously fit spectra corresponding to different metasurface scaling factors with shared parameters (Fig. S4a). The exciton position ω_{Ex} is fixed at 476.6 THz and its γ_{Ex} at 8.7 THz, as reported (*44*). The uncoupled qBIC dispersion is approximated as a linear function, with its slope and offset treated as shared fit parameters. $\gamma_{\mathrm{qBIC,int}}$ is directly relatable to the material dispersion in the absence of no exciton (see Fig. S1b). Due to this increased material absorption at smaller scaling factors, γ_{qBIC} increases, which is also modeled as a linear function of the scaling factor (Fig. S4b). We evaluate the coupling strength $\kappa = 13.07$ THz at the scaling factor $S \approx 1.035$, where the qBIC and exciton resonances overlap, and calculate the generalized Rabi frequency as

$$\Omega_R = 2\sqrt{\kappa^2 - \frac{(\gamma_{\mathrm{qBIC}} - \gamma_{\mathrm{Ex}})^2}{4}}. \tag{S25}$$

The polariton branches are described by

$$\omega_{\pm} = \frac{\omega_{\mathrm{qBIC}} + \omega_{\mathrm{Ex}}}{2} + i\frac{\gamma_{\mathrm{qBIC}} + \gamma_{\mathrm{Ex}}}{2} \pm \sqrt{\kappa^2 - \frac{1}{4}\left(\gamma_{\mathrm{qBIC}} - \gamma_{\mathrm{Ex}} + i(\omega_{\mathrm{qBIC}} - \omega_{\mathrm{Ex}})\right)^2}, \tag{S26}$$

which shows excellent agreement with our experimental transmittance spectra (Fig. S4c).

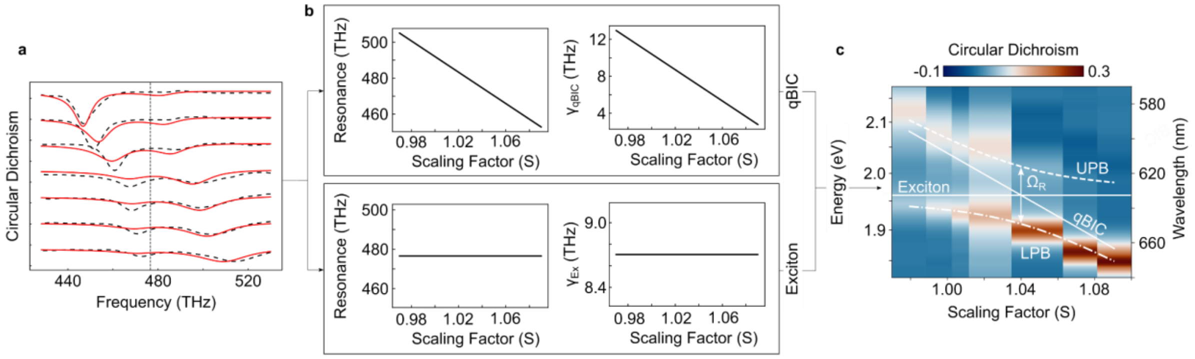

Fig. S4. Temporal coupled-mode theory for BIC–exciton coupling.

(A) Simultaneous TCMT fit of metasurfaces with multiple scaling factors. **(B)** Scaling factor dependence of resonance position and damping rate. The BIC dispersion is linear for both the resonance position and damping rate (upper panel), while the exciton position and damping rate remain constant (lower panel). **(C)** Branches of strongly coupled exciton-polaritons. The colormap shows the circular dichroism from experimental transmittance spectra, where the overlayed polariton branches are calculated from above fit parameters, showing excellent agreement, corroborating the accuracy of our fit approach.

Supplementary Note 6: Strong Coupling Fits in k-Space

To further corroborate the validity of our TCMT fits, we conduct additional fits of experimental reflectance spectra for different scaling factors in the k-space. We estimate the upper limit of the Rabi splitting $\Omega_R$by using a simplified Hamiltonian

$$\Omega = \begin{pmatrix} \omega_{\mathrm{qBIC}} & \kappa \\ \kappa & \omega_{\mathrm{Ex}} \end{pmatrix}, \tag{S27}$$

which leads to the following simplified polariton dispersions

$$\omega_{\pm}(k) = \frac{\omega_{\mathrm{qBIC}}(k)+\omega_{\mathrm{Ex}}}{2} \pm \sqrt{\kappa^2 + \frac{1}{4}\left(\omega_{\mathrm{qBIC}}(k) - \omega_{\mathrm{Ex}}\right)^2}. \tag{S28}$$

While in simulation, the dispersion of the qBIC $\omega_{\mathrm{qBIC}}(k)$ is known and used to fit the polariton branches, in experiment, we approximate the dispersion with the quadratic relation

$$\omega_{\mathrm{qBIC}}(k) = Ak^2 + U(S), \tag{S29}$$

with A and C being fit parameters. We further assume that the parameter *A* is the same for all scale factors probed and that *C* follows a linear relationship with the scaling factor *S*:

$$U(S) = U_1 * S + U_2.$$

Similarly to the above fit of multiple spectra with shared fit parameters, we share the parameter *A* as well as the slope and offset of the parameter *U* over a scaling factor range from *S* = 0.95 to *S* = 1.11. Fixing the coupling strength κ to 13.07 THz, taken from the above TCMT fits, yields excellent agreement of the polariton fits with the strongly coupled experimental data (Fig. S5).

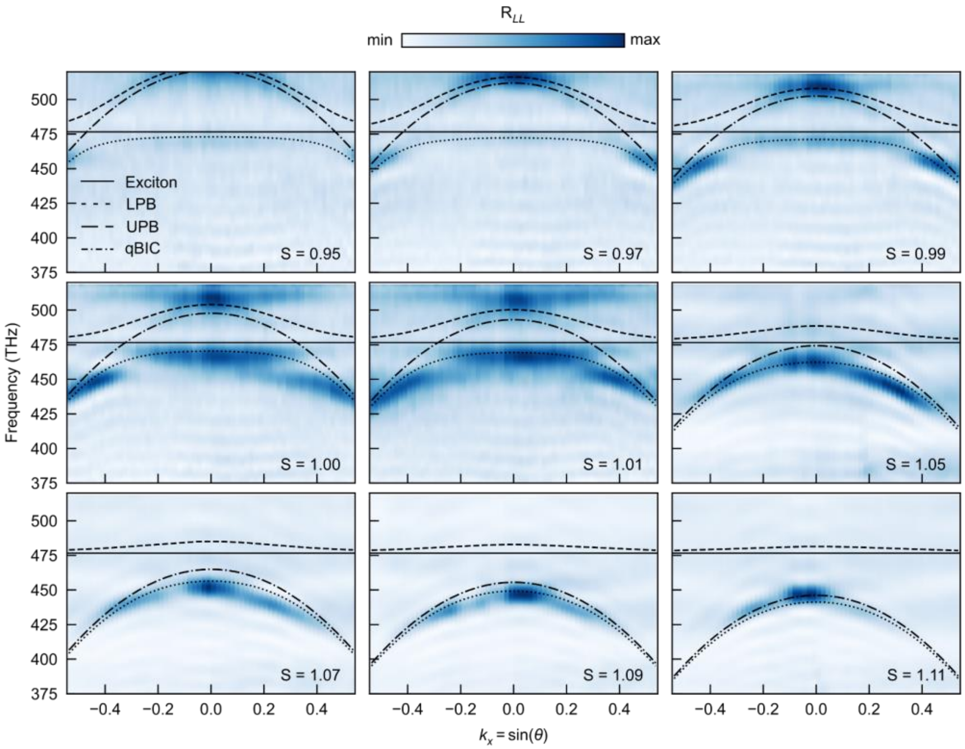

Fig. S5. Experimental k-space polariton fits.
Upper- and lower polariton dispersions fitted to experimental angle-resolved reflectance spectra for left-circularly polarized light (R_{LL}) with a fixed coupling strength $\kappa$and parabolic dispersion of the quasi-BIC mode, which is linearly shifted with the scaling factor *S*.

Supplementary Note 7: Hopfield Coefficients

To quantify the ratio between excitonic and photonic modes in the resulting polaritons, we express the polaritonic eigenstates as linear combinations of the qBIC $|qBIC\rangle$ and exciton $|Ex\rangle$ modes:

$$|LP\rangle = X_{LP}|Ex\rangle + B_{LP}|qBIC\rangle, \qquad |UP\rangle = X_{UP}|Ex\rangle + B_{UP}|qBIC\rangle,$$

where X_{LP}, X_{UP}, B_{LP} and B_{UP} are the Hopfield coefficients with:

$$|X_{LP}|^2 = |B_{UP}|^2 = |C_-|^2, \qquad |X_{UP}|^2 = |B_{LP}|^2 = |C_+|^2,$$

and

$$|C_+|^2 = \frac{1}{2}\left(1 + \frac{\Delta}{\sqrt{\Delta^2 + \Omega_R^2}}\right), \qquad |C_-|^2 = \frac{1}{2}\left(1 - \frac{\Delta}{\sqrt{\Delta^2 + \Omega_R^2}}\right).$$

Here $\Delta = \omega_{Ex} - \omega_{qBIC}$ is the detuning and $\Omega_R = 26$ THz is the Rabi splitting extracted from our experimental data.

We plot the Hopfield coefficients as functions of both scaling factor S (Fig. S6a) and in-plane momentum k_x/k_0 for S=0.99 (Fig. S6c), using TCMT fitting of the experimental spectra (Figs. S6b and d). In both cases, the lower values of the Hopfield coefficients do not fall below 0.1, indicating that even in the most detuned conditions, the polaritons cannot be treated as purely photonic or excitonic modes. Moreover, even a small qBIC fraction is sufficient to impart circular polarization onto the polariton mode.

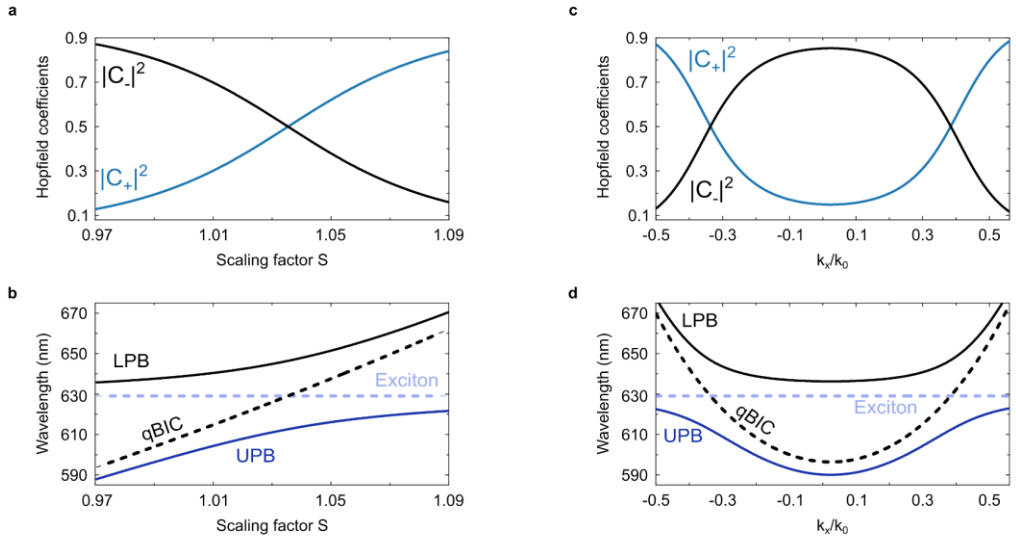

Fig. S6. Estimation of Hopfield Coefficients
(A) Hopfield coefficients as functions of scaling factor S, calculated using (**B**) data extracted from TCMT fitting of experimental data. (**C**) and (**D**) Corresponding results in momentum space for the strong coupling, shown for the metasurface with S=0.99.

Supplementary Note 8: Linear Chirality in Strong and Weak Coupling Regimes

To transition from the strong to the weak coupling regime, the inequality between the coupling strength κ and the losses of qBIC γ_{qBIC} and excitons γ_{Ex} must satisfy:

$$\kappa < \frac{|\gamma_{qBIC} - \gamma_{Ex}|}{2}$$

Initially, our system operates in the regime of maximized Rabi splitting, where $\gamma_{\mathrm{qBIC}} \approx \gamma_{\mathrm{Ex}}$ and κ = 13.1 THz (obtained from TCMT fitting). Under these conditions, it is practically impossible to reach the weak coupling regime solely by tuning γ_{BIC} while maintaining maximum chirality.

Therefore, we fixed $\gamma_{Ex} = 8.7$ THz, decreased γ_{qBIC} by altering the metasurface geometric parameters and set $\kappa = 13.1\beta$ THz, with a variable dimensionless factor $0 \leq \beta \leq 1$. The designed new maximally chiral metasurface with a higher Q-factor has the following structure parameters for S = 1: a = 220 nm, b = 90 nm, c = 65 nm, $L_x = L_y$=340 nm, and $\alpha = 5°$. In the absence of exciton ($\beta = 0$, Fig.S7b) $\gamma_{qBIC} = 4.4$ THz.

By setting $\beta = 1/7$ (Fig.S7c) we decreased the coupling strength of the qBIC and excitons to establish the weak coupling regime. Finally, setting $\beta = 1$ allows us to study the same metasurface in the strong coupling regime analogous to that in the main text (Fig.S7d).

The resulting transmittance difference spectra $\Delta T = T_{RR} - T_{LL}$ for the metasurface with no excitons ($\beta = 0$), in the weak ($\beta = 1/7$) and strong coupling ($\beta = 1$) regimes are shown in Fig.S7b-d. As we can see, in the weak coupling regime it is impossible to resolve the Rabi splitting, whereas in the strong coupling regime polariton branch with smaller wavelengths become barely distinguishable due to strong excitonic absorption and the high Q-factor of the qBIC. Furthermore, the linear chiral properties of the metasurface in the weak coupling regime largely resemble those of the system without excitons.

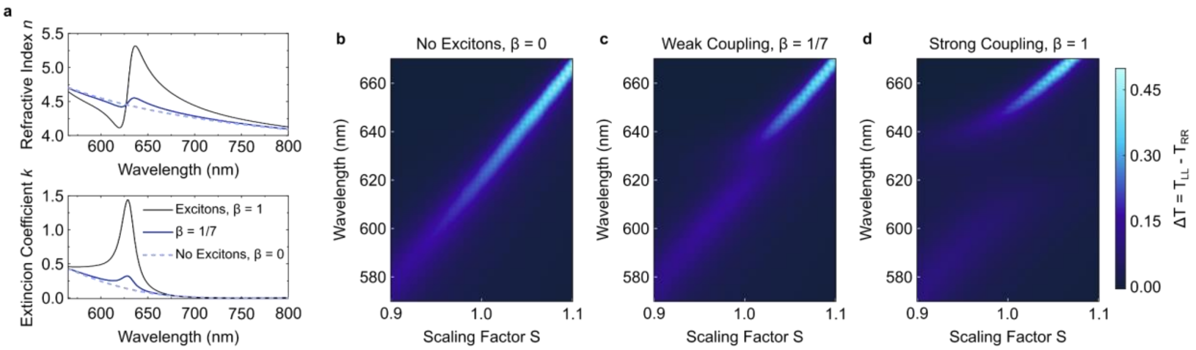

Fig. S7. Linear optical chirality in weak and strong coupling regimes

(A) Dispersion of the refractive index *n* and extinction coefficient *k* for bulk WS_2 with different coupling strength $\kappa = 13.1\beta$ THz, where $\beta = 1$ corresponds to the tabulated dispersion (*59*) and $\beta = 0$ to the absence of excitonic resonance. Transmittance differences $\Delta T = T_{RR} - T_{LL}$ for a metasurface with a = 220 nm, b = 90 nm, c = 65 nm, $L_x = L_y$=340 nm, and $\alpha = 5°$ (S=1), operating in **(B)** the absence of excitons **(C)** weak coupling and **(D)** strong coupling regimes.

Supplementary Note 9: THG from Unpatterned 105 nm Thick WS2 Flake

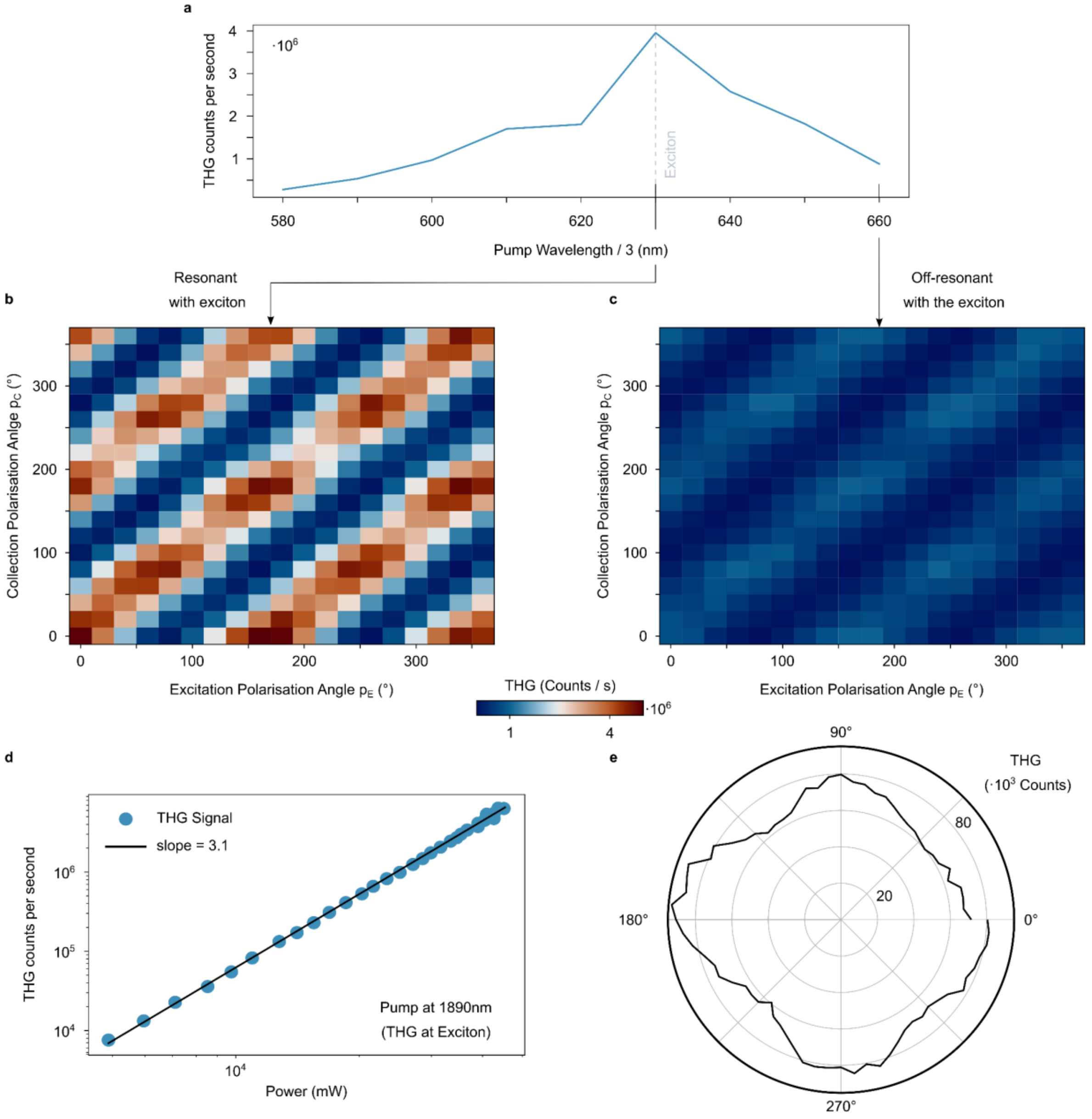

Fig. S8. THG of unpatterned 105 nm thick WS_2 Flake excited with linearly polarized light **(A)** THG counts per second vs. pump wavelength/3, showing intrinsic enhancement of nonlinear generation near the exciton. **(B)** Polarization dependence of resonant THG (pump: 1890 nm, 15 mW) reveals no preferred polarization in unpatterned material. **(C)** Polarization dependence of off-resonant THG (pump: 1980 nm, 15 mW) similarly shows no polarization preference, with reduced THG confirming exciton-enhanced generation. **(D)** Power dependence of resonant THG from unpatterned WS_2 shows a slope of 3.1 on a double-log scale. **(E)** Normalized polarization dependent THG from unpatterned WS_2 at 1860 nm, 15 mW pump.

Supplementary Note 10: THG from WS_2 Metasurfaces Excited with Linearly Polarized Light

We characterized the power dependence of the nonlinear signal to verify the order of the generated harmonic. To this end, we plotted the signal intensity as a function of pump power on a double-logarithmic scale (Fig. S7). The slope of the linear fit to this data determines the harmonic order. The extracted slope closely matches 3, confirming that the signal arises from a third-order process (in the present case THG). Next, we examined the influence of the qBIC metasurface by sweeping the excitation polarization and comparing the THG response from both patterned and unpatterned WS_2 (Fig. S7b). In the x-y plane, the unpatterned WS_2 is isotropic (with anisotropy only along the z-axis), resulting in no preferred polarization for the THG signal. The polarization response of the unpatterned sample therefore forms a circle. In contrast, the polarization-dependent THG from the qBIC metasurface exhibits a pronounced dumbbell shape, indicating a strong polarization dependence. The maximum THG signal aligns with the preferred excitation polarization of the qBIC mode in the double-rod unit cell (parallel to the principal axes), confirming that the THG is mediated by the qBIC. When the excitation polarization is orthogonal to the BIC mode - where the qBIC resonance vanishes - the THG signal nearly disappears, providing further evidence that the nonlinear response is governed by the qBIC. For further confirmation and to get a more comprehensive picture, the THG is swept for the entire parameter space spanned by the excitation and collection polarizations (p_E and p_C, respectively) (Fig. S7d). The data again indicates that there is clear preferred excitation polarization that maximizes THG, which in turn is consistent with the polarization that leads to the strongest qBIC formation.

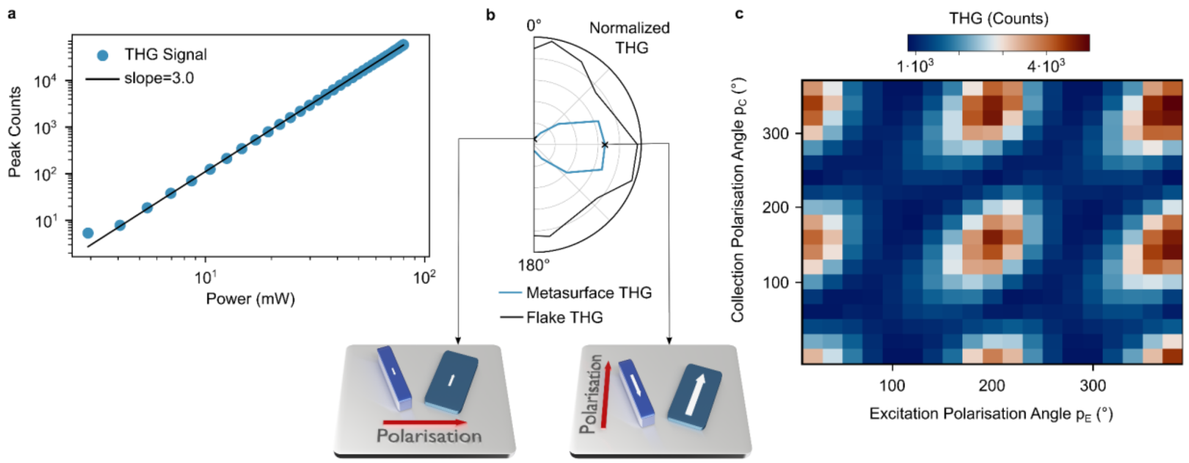

Fig. S9. THG under linearly polarized illumination.
(A) The power dependence of the higher harmonic signal from the chiral WS_2 metasurfaces shows a linear trend with a slope of 3 when plotted on a double log-scale, typical for third harmonic signal. **(B)** Normalized polarization dependent THG signal from the WS_2 metasurface excited with linearly polarized light. The metasurface-enhanced THG signal shows a characteristic dumbbell shape that is absent from the unpatterned WS_2, highlighting the importance of the qBIC resonance for THG generation in the structure. The respective lines do not close due to the beam shift caused by changing the polarization. **(C)** Influence of excitation and collection polarization on the THG signal.

Supplementary Note 11: Polarization Analysis of THG from WS_2 Metasurfaces

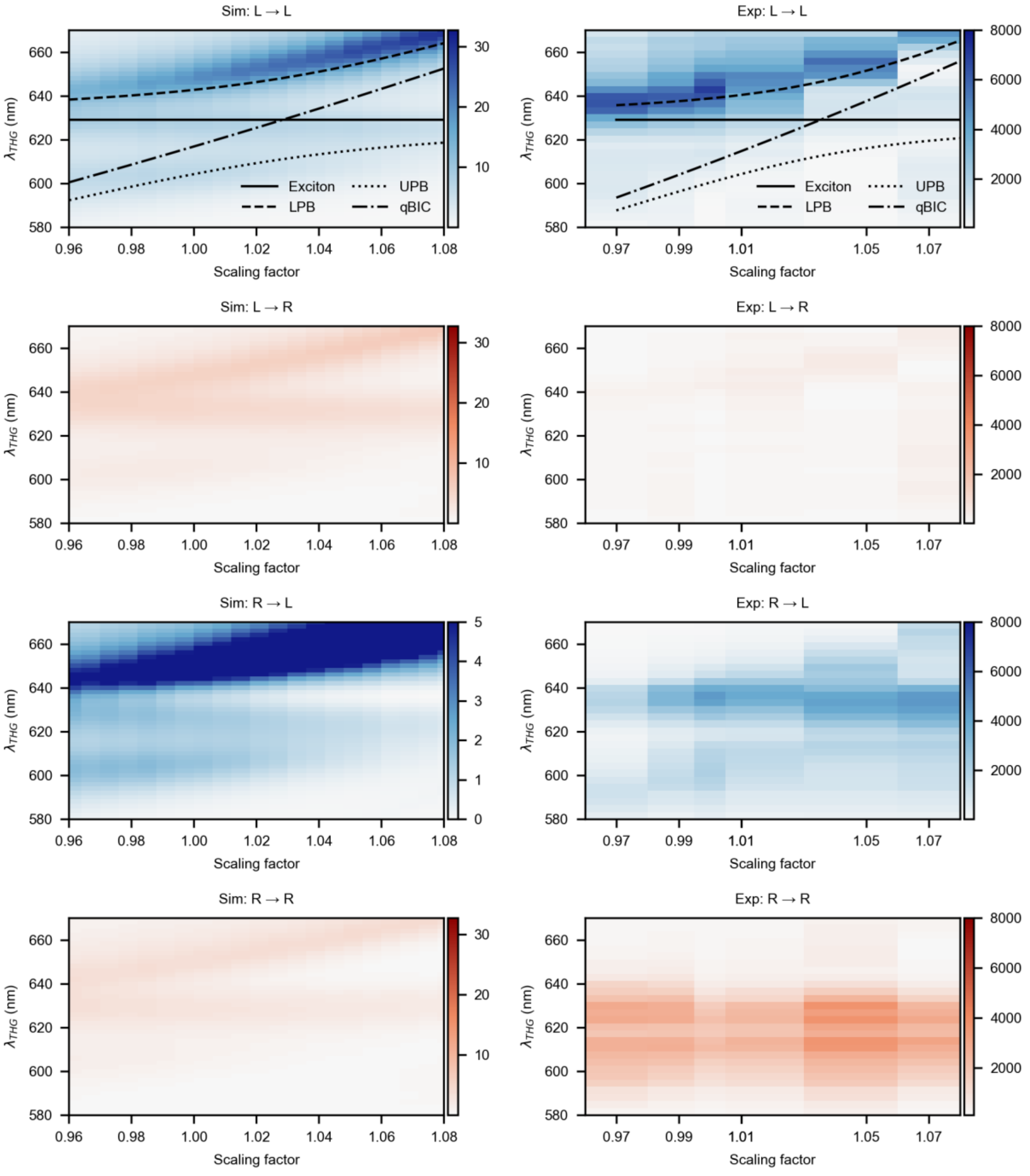

Fig. S10. THG chiral pump polarization.

THG spectra in counts per seconds (experiment) and arbitrary units (simulations) for different polarizations of excitation. For left-polarized pump and left-polarized THG, the experimental map is overlaid with the upper and lower polariton branch (UPB/LPB) dispersions obtained from TCMT fit of the corresponding linear (ω) measurements acquired on the same sample (Main Text Fig. 3A). Analogously, the simulated THG map is overlaid with polariton dispersions extracted from the corresponding linear simulations. We attribute the small deviation in the

polariton position in linear and nonlinear regimes to an additional resonant $|\chi^3|$ contribution (Main Text Fig. 4B)

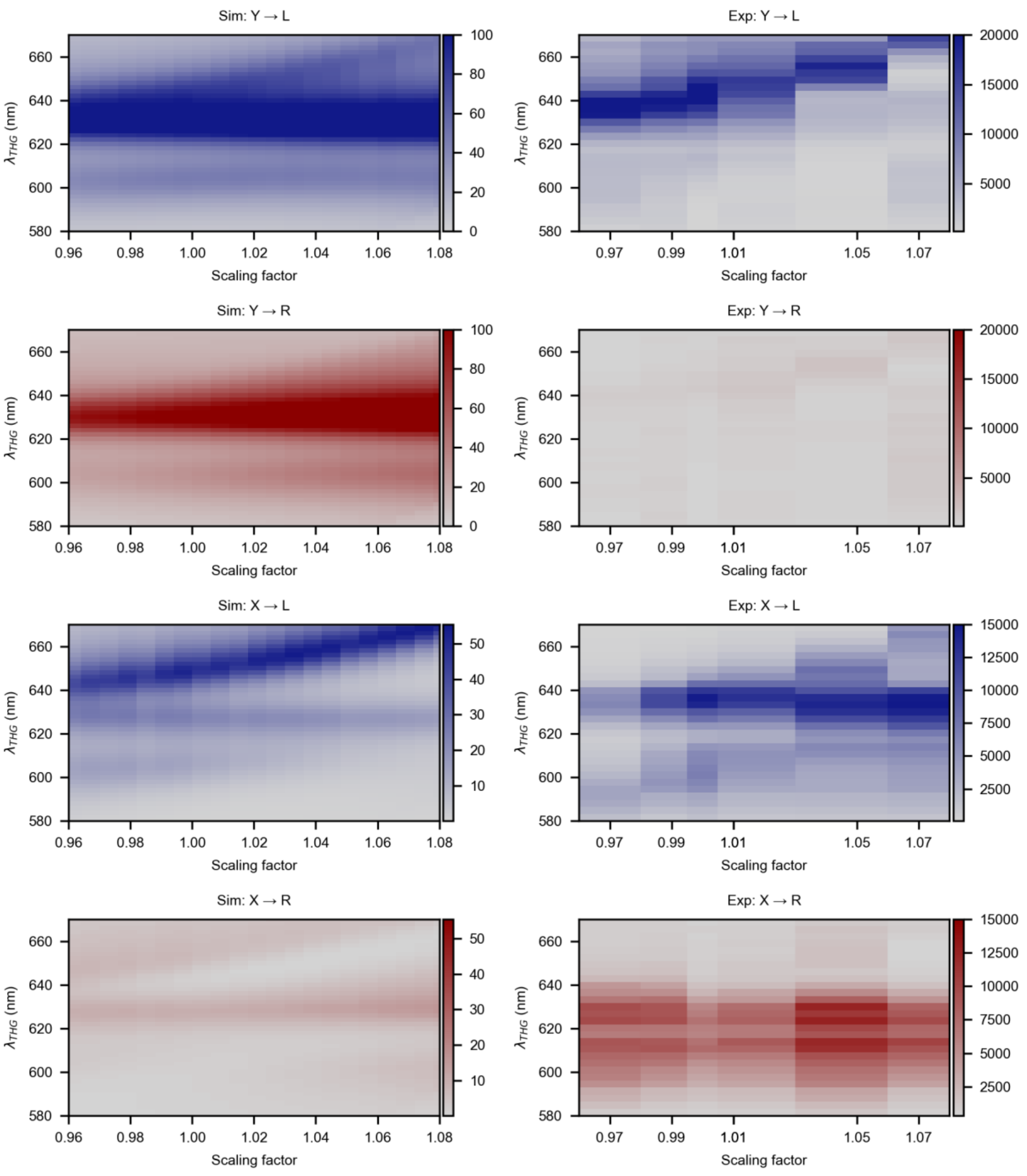

Fig. S11. THG linear pump polarization.
THG spectra in counts per seconds (experiment) and arbitrary units (simulations) for different polarizations of excitation.

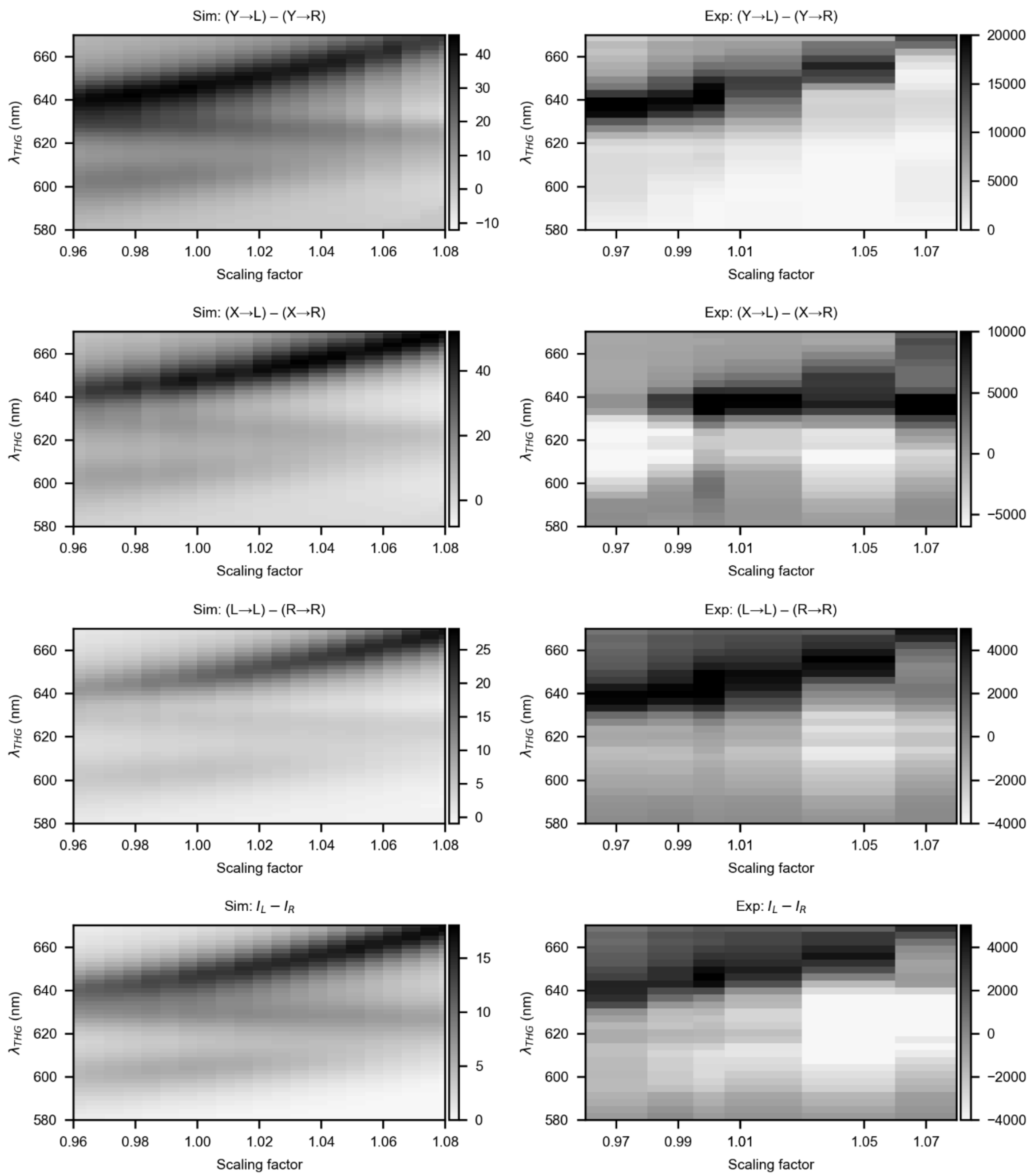

Fig. S12. THG intensity differences

THG spectra in counts per second (experiment) and arbitrary units (simulations) for different polarizations of excitation. I_L (I_R) is the total THG intensity collected for LCP (RCP) pumping: $I_L = L \rightarrow L + L \rightarrow R$ ($I_R = R \rightarrow R + R \rightarrow L$).

Supplementary Note 12: Experimental THG Efficiency

THG efficiencies (Fig. S11) were estimated by measuring the detection efficiency with a calibrated light source for two selected wavelengths (500 nm and 666 nm) and interpolating between them. This does not include losses induced by the finite solid collection angle of the transmission objective. Therefore, the values can be interpreted as lower bound for the absolute, angle-integrated efficiencies.

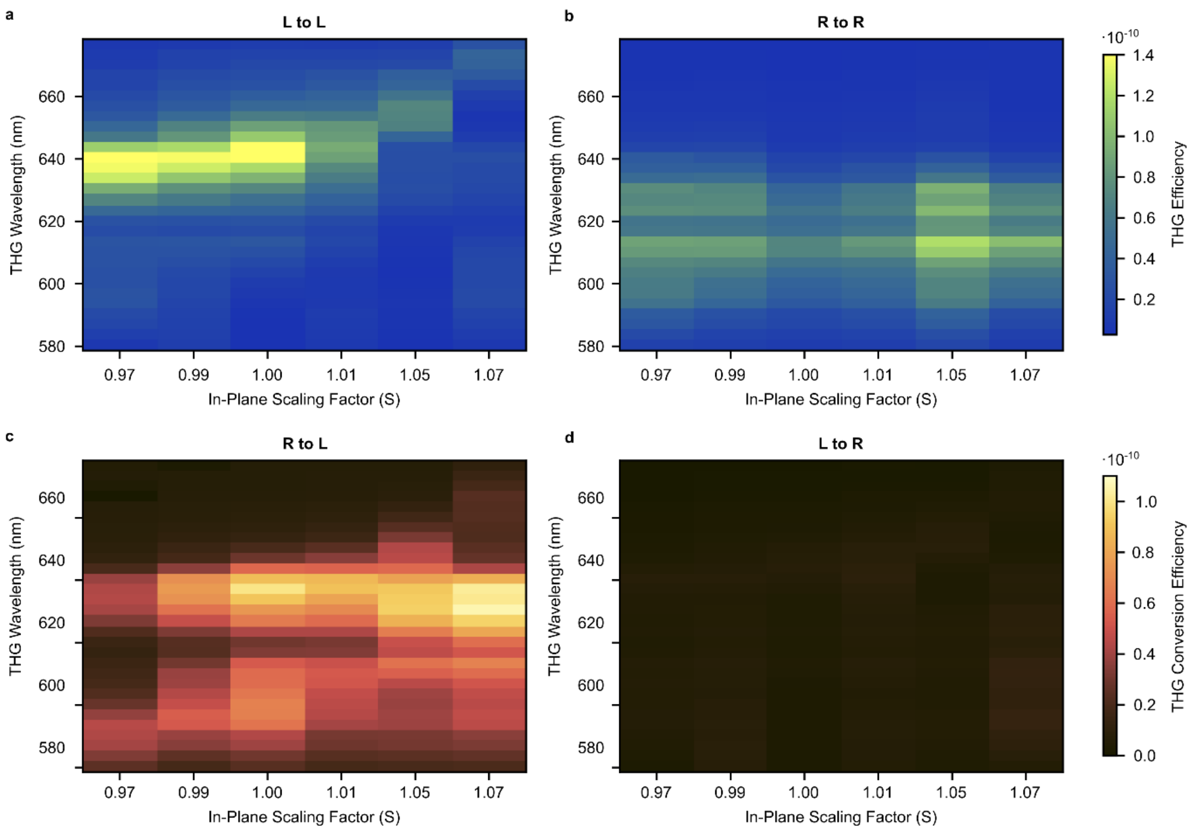

Fig. S13. Experimental THG efficiency

(A) THG efficiency for L to L and **(B)** R to R. (C) THG conversion efficiency for R to L and **(D)** L to R, at an excitation power of 50 mW.

Supplementary Note 13: THG in Strong and Weak Coupling Regimes

We use the metasurface from Fig.S7 and investigate its nonlinear properties in both weak ($\beta = 1/7$) and strong ($\beta = 1$) coupling regimes. Since γ_{Ex}=8.7 THz is fixed, we can use the extracted $|\chi^{(3)}|$ function from Fig.4b of the main text to compare the right- and left-handed signals. The simulated THG data for the new metasurface with $\beta = 1$ and $\beta = 1/7$ are presented in Fig. S15.

All key results for the strong coupling regime remain consistent with those in the main text: chiral polaritons inherit circular polarization from the qBIC and gain enhancement from the excitons, leading to LCP THG regardless of the pump polarization. In the RCP signal we observe THG with only excitonic enhancement at wavelengths around λ_{Ex}.

For the weak coupling regime, the situation is apparently different. No polariton branches are formed, and one can interpret the qBIC role as that of a chiral cavity: it enhances the excitonic THG when crossing the exciton band $\lambda_{Ex}=\lambda_{qBIC}$ (S = 1) and introduces a chiral dissymmetry as LCP harmonics is emitted somewhat stronger than RCP. For better clarity, we plot the TH intensity spectra for a particular metasurface with $S = 1$ in Fig. S15c and d for the strong and weak coupling regimes respectively. One can see that in the weak coupling regime the excitons at $\lambda_{Ex} \approx 629$ nm emit a chiral signal. In the strong coupling regime, on the contrary, the signals L to L and R to R are almost identical at about λ_{Ex}. The difference between “left” and “right” appears at the wavelengths of chiral polaritons $\lambda \approx 605$ nm and $\lambda \approx 650$ nm.

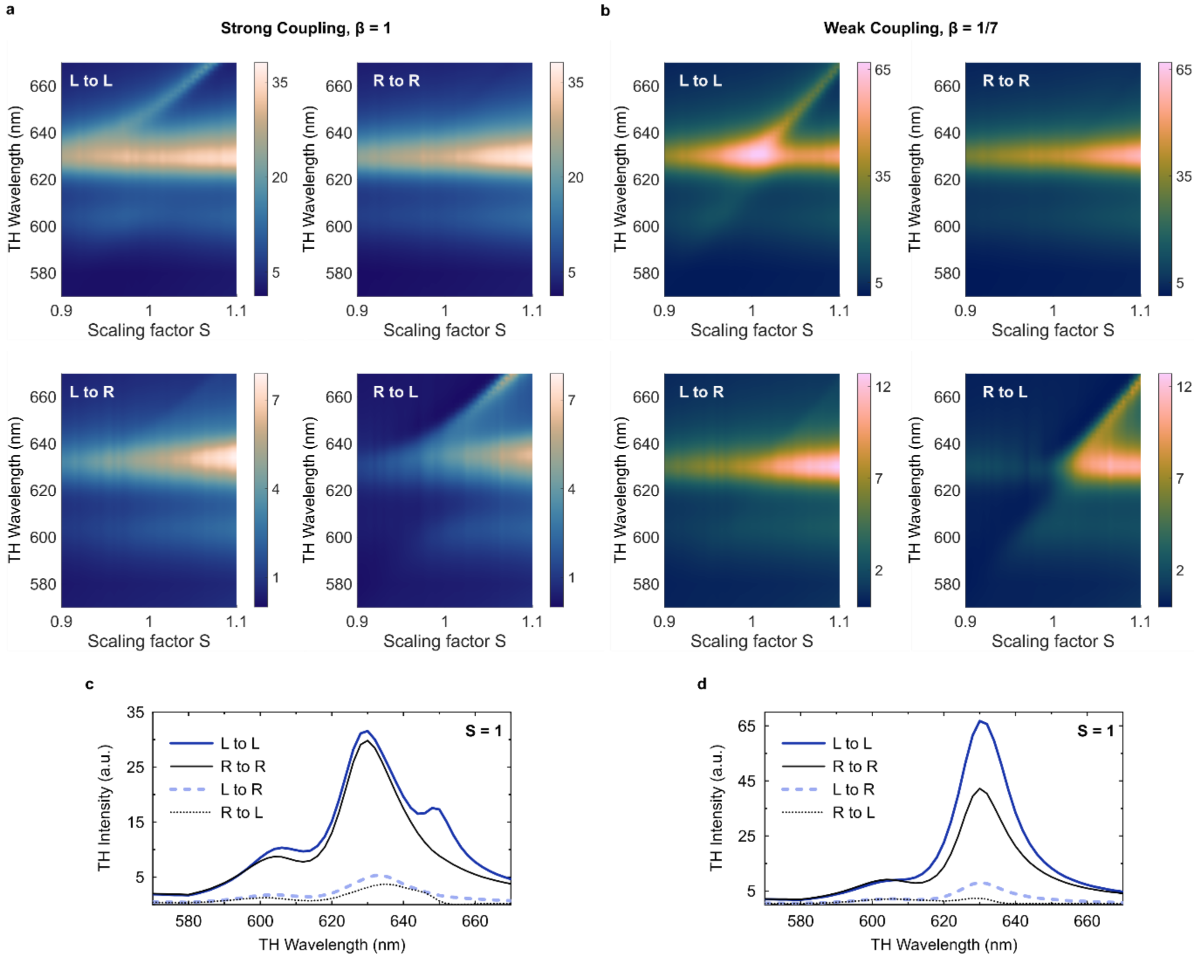

Fig. S14. Nonlinear optical chirality in weak and strong coupling regimes.
TH intensities in a.u. as functions of the TH wavelength and scaling factor S for the metasurface operating in **(A)** the strong ($\beta = 1$) and **(B)** the weak ($\beta = 1/7$) coupling regimes. The pump (P) and TH (H) polarizations are indicated as P to H labels. TH intensities spectra at scaling factor S = 1 for **(C)** $\beta = 1$ and **(D)** $\beta = 1/7$, respectively.

Supplementary Note 14: Sketches of Measurement Setups

A sketch of the chiral measurement setup is shown below. The excitation objective was switched to 20x magnification for the measurements at oblique angle of incidence to improve possible alignment quality.

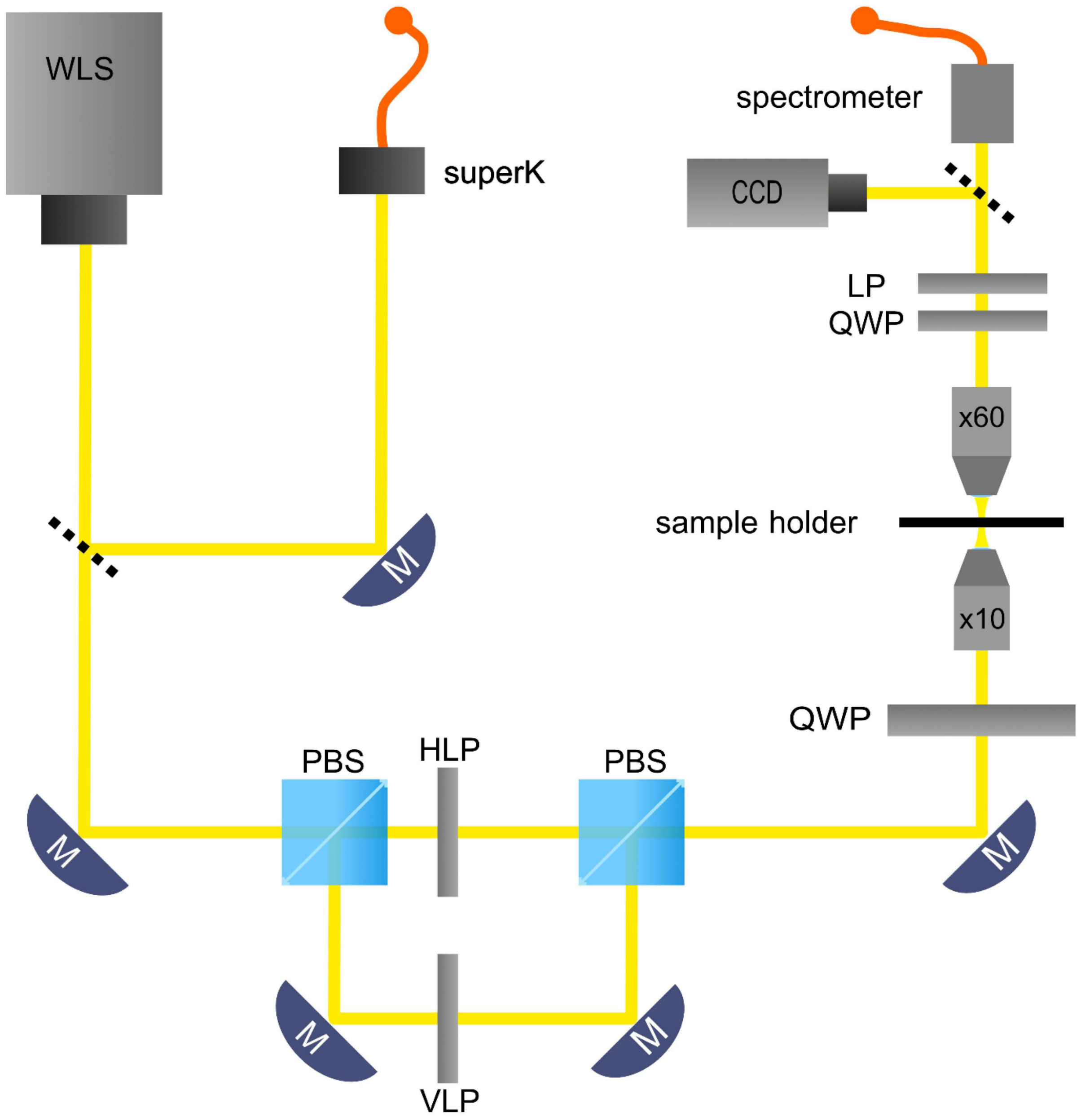

Fig. S15. Sketch of chiral measurement setup.
Used abbreviations are WLS: white light source, M: mirror, FM: flip mirror, PBS: polarizing beam splitter, HLP (VLP): horizontal (vertical) linear polarizer, QWP: quarter-wave plate, LP: linear polarizer.

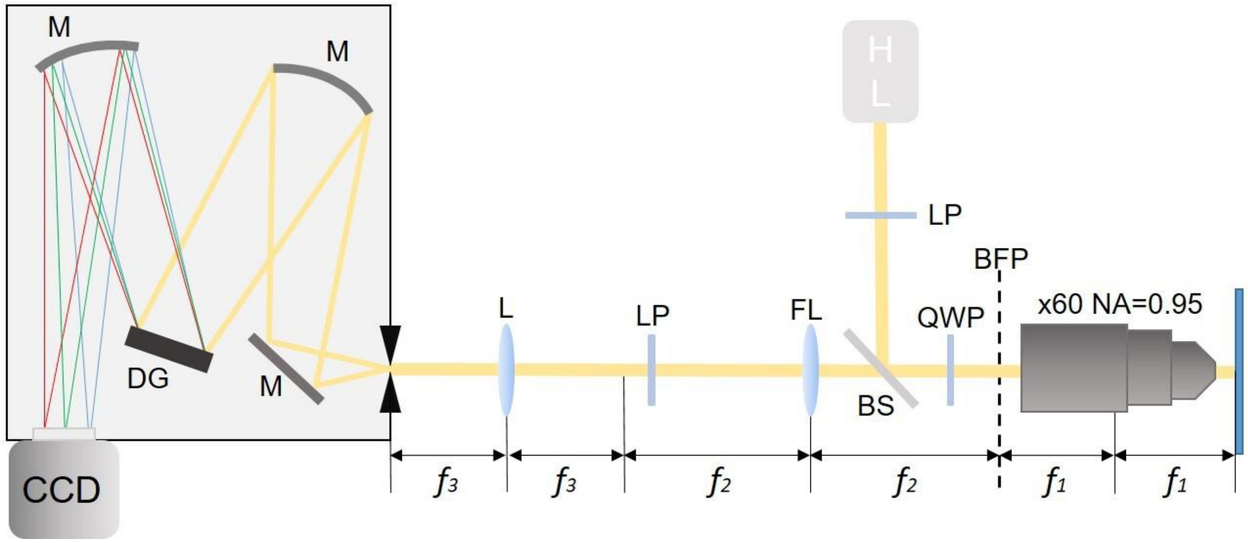

Fig. S16. Sketch of back focal plane spectroscopy setup
Schematic of the k-space angle-resolved reflection setup. A halogen lamp (HL) provides collimated white light, which is circularly polarized via a linear polarizer (LP) and quarter-wave plate (QWP) before being directed to the sample through a 60×, 0.95 NA objective via a 50:50 beamsplitter (BS). Reflected light, collected by the same objective, passes back through the QWP and an analyzer LP to resolve polarization states. A Fourier lens (FL) images the back focal plane (BFP), and a 4-f system relays the image onto the entrance slit of a spectrometer. The spectrally dispersed signal (M: mirror; DG: diffraction grating) is then recorded by a CCD camera.

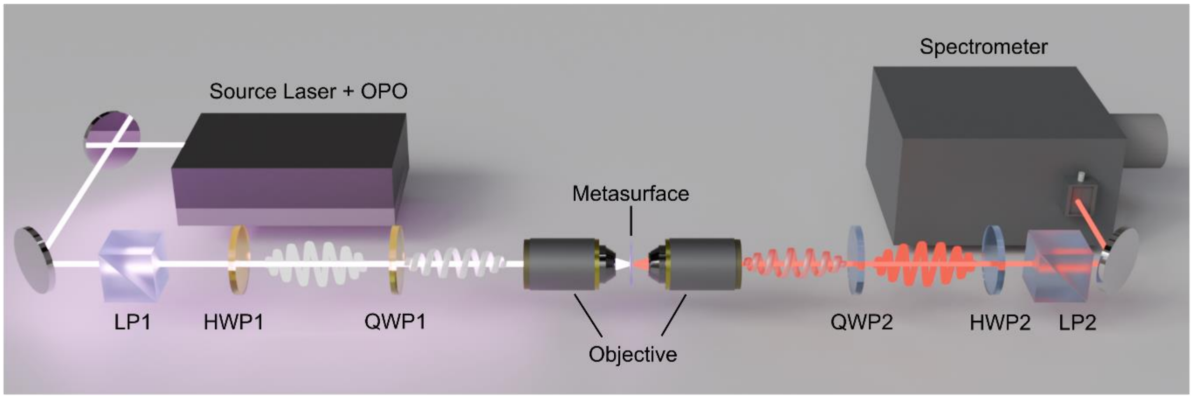

Fig. S17. Sketch of chiral nonlinear measurements setup.
For linear THG measurements, the QWPs are removed. LP1 and LP2: Glan-Taylor-Prisms, HWP1 and QWP1: waveplates for the infrared, QWP2 and HWP2: visible waveplates.

REFERENCES

1. C. Zhang, H. Hu, C. Ma, Y. Li, X. Wang, D. Li, A. Movsesyan, Z. Wang, A. Govorov, Q. Gan, T. Ding, Quantum plasmonics pushes chiral sensing limit to single molecules: A paradigm for chiral biodetections. *Nat. Commun.* **15**, 2 (2024).

2. J. Garcia-Guirado, M. Svedendahl, J. Puigdollers, R. Quidant, Enhanced chiral sensing with dielectric nanoresonators. *Nano Lett.* **20**, 585–591 (2019).

3. X. Li, A. C. Jones, J. Choi, H. Zhao, V. Chandrasekaran, M. T. Pettes, A. Piryatinski, M. A. Tschudin, P. Reiser, D. A. Broadway, P. Maletinsky, N. Sinitsyn, S. A. Crooker, H. Htoon, Proximity-induced chiral quantum light generation in strain-engineered WSe_2/$NiPS_3$ heterostructures. *Nat. Mater.* **22**, 1311–1316 (2023).

4. R. Shreiner, K. Hao, A. Butcher, A. A. High, Electrically controllable chirality in a nanophotonic interface with a two-dimensional semiconductor. *Nat. Photonics* **16**, 330–336 (2022).

5. M. Claassen, C. Jia, B. Moritz, T. P. Devereaux, All-optical materials design of chiral edge modes in transition-metal dichalcogenides. *Nat. Commun.* **7**, 13074 (2016).

6. G. Wang, A. Chernikov, M. M. Glazov, T. F. Heinz, X. Marie, T. Amand, B. Urbaszek, Colloquium: Excitons in atomically thin transition metal dichalcogenides. *Rev. Mod. Phys.* **90**, 021001 (2018).

7. P. Chen, T. W. Lo, Y. Fan, S. Wang, H. Huang, D. Lei, Chiral coupling of valley excitons and light through photonic spin–orbit interactions. *Adv. Opt. Mater.* **8**, 1901233 (2020).

8. N. Zibouche, P. Philipsen, A. Kuc, T. Heine, Transition-metal dichalcogenide bilayers: Switching materials for spintronic and valleytronic applications. *Phys. Rev. B* **90**, 125440 (2014).

9. J. Lee, S. Kang, K. Yim, K. Y. Kim, H. W. Jang, Y. Kang, S. Han, Hydrogen evolution reaction at anion vacancy of two-dimensional transition-metal dichalcogenides: Ab initio computational screening. *J. Phys. Chem. Lett.* **9**, 2049–2055 (2018).

10. D. Varsano, M. Palummo, E. Molinari, M. Rontani, A monolayer transition-metal dichalcogenide as a topological excitonic insulator. *Nat. Nanotechnol.* **15**, 367–372 (2020).

11. W. Shi, J. Ye, Y. Zhang, R. Suzuki, M. Yoshida, J. Miyazaki, N. Inoue, Y. Saito, Y. Iwasa, Superconductivity series in transition metal dichalcogenides by ionic gating. *Sci. Rep.* **5**, 12534 (2015).

12. C. Schneider, M. M. Glazov, T. Korn, S. Höfling, B. Urbaszek, Two-dimensional semiconductors in the regime of strong light-matter coupling. *Nat. Commun.* **9**, 2695 (2018).

13. S. Kim, Y. Jang, D. Han, J. Lee, J. Rho, Van der Waals metasurfaces molding topological polaritons. *Rev. Phys.* **13**, 100115 (2025).

14. D. Sanvitto, S. Kéna-Cohen, The road towards polaritonic devices. *Nat. Mater.* **15**, 1061–1073 (2016).

15. S. Ghosh, T. C. Liew, Quantum computing with exciton-polariton condensates. *npj Quantum Inf.* **6**, 16 (2020).

16. J.-W. Kang, B. Song, W. Liu, S.-J. Park, R. Agarwal, C.-H. Cho, Room temperature polariton lasing in quantum heterostructure nanocavities. *Sci. Adv.* **5**, eaau9338 (2019).

17. S. Dufferwiel, S. Schwarz, F. Withers, A. A. Trichet, F. Li, M. Sich, O. Del Pozo- Zamudio, C. Clark, A. Nalitov, D. D. Solnyshkov, G. Malpuech, K. S. Novoselov, J. M. Smith, M. S. Skolnick, D. N. Krizhanovskii, A. I. Tartakovskii, Exciton–polaritons in van der Waals heterostructures embedded in tunable microcavities. *Nat. Commun.* **6**, 8579 (2015).

18. D. G. Baranov, C. Schäfer, M. V. Gorkunov, Toward molecular chiral polaritons. *ACS Photonics* **10**, 2440–2455 (2023).

19. Q. Cheng, J. Yang, L. Sun, C. Liu, G. Yang, Y. Tao, X. Sun, B. Zhang, H. Xu, Q. Zhang, Tuning the plexcitonic optical chirality using discrete structurally chiral plasmonic nanoparticles. *Nano Lett.* **23**, 11376–11384 (2023).

20. J. Zhu, F. Wu, Z. Han, Y. Shang, F. Liu, H. Yu, L. Yu, N. Li, B. Ding, Strong light–matter interactions in chiral plasmonic–excitonic systems assembled on DNA origami. *Nano Lett.* **21**, 3573–3580 (2021).

21. P. E. Stamatopoulou, S. Droulias, G. P. Acuna, N. A. Mortensen, C. Tserkezis, Reconfigurable chirality with achiral excitonic materials in the strong-coupling regime. *Nanoscale* **14**, 17581–17588 (2022).

22. X. Wang, E. Ronca, M. A. Sentef, Cavity quantum electrodynamical Chern insulator: Towards light-induced quantized anomalous Hall effect in graphene. *Phys. Rev. B* **99**, 235156 (2019).

23. A. Frisk Kockum, A. Miranowicz, S. De Liberato, S. Savasta, F. Nori, Ultrastrong coupling between light and matter. *Nat. Rev. Phys.* **1**, 19–40 (2019).

24. H. Hübener, U. De Giovannini, C. Schäfer, J. Andberger, M. Ruggenthaler, J. Faist, A. Rubio, Engineering quantum materials with chiral optical cavities. *Nat. Mater.* **20**, 438–442 (2021).

25. K. Voronin, A. S. Taradin, M. V. Gorkunov, D. G. Baranov, Single-handedness chiral optical cavities. *ACS Photonics* **9**, 2652–2659 (2022).

26. N. Yu, F. Capasso, Flat optics with designer metasurfaces. *Nat. Mater.* **13**, 139–150 (2014).

27. J. Valentine, S. Zhang, T. Zentgraf, E. Ulin-Avila, D. A. Genov, G. Bartal, X. Zhang, Three-dimensional optical metamaterial with a negative refractive index. *Nature* **455**, 376–379 (2008).

28. X. Yin, Z. Ye, J. Rho, Y. Wang, X. Zhang, Photonic spin Hall effect at metasurfaces. *Science* **339**, 1405–1407 (2013).

29. K. Koshelev, A. Bogdanov, Y. Kivshar, Meta-optics and bound states in the continuum. *Sci. Bull.* **64**, 836–842 (2019).

30. M. V. Gorkunov, A. A. Antonov, Y. S. Kivshar, Metasurfaces with maximum chirality empowered by bound states in the continuum. *Phys. Rev. Lett.* **125**, 093903 (2020).

31. H. Zhou, M. Qin, H. Xu, G. Wei, H. Li, W. Gao, J. Liu, F. Wu, Photonic spin-controlled self-hybridized exciton-polaritons in WS_2 metasurfaces driven by chiral quasibound states in the continuum. *Phys. Rev. B* **109**, 125201 (2024).

32. F. J. Löchner, A. George, K. Koshelev, T. Bucher, E. Najafidehaghani, A. Fedotova, D.-Y. Choi, T. Pertsch, I. Staude, Y. Kivshar, A. Turchanin, F. Setzpfandt, Hybrid dielectric metasurfaces for enhancing second-harmonic generation in chemical vapor deposition grown MoS_2 monolayers. *ACS Photonics* **8**, 218–227 (2021).

33. J. Lee, M. Jeong, J. Jang, J. Kim, J. Mun, X. Gong, R. Fang, Y. Yang, S. H. Chae, S. Kim, J. Rho, Bound-states-in-the-continuum-induced directional photoluminescence with polarization singularity in WS_2 monolayers. *Nano Lett.* **25**, 861–867 (2025).

34. L. Sortino, M. Brooks, P. G. Zotev, A. Genco, J. Cambiasso, S. Mignuzzi, S. A. Maier, G. Burkard, R. Sapienza, A. I. Tartakovskii, Dielectric nanoantennas for strain engineering in atomically thin two-dimensional semiconductors. *ACS Photonics* **7**, 2413–2422 (2020).

35. Y. Pu, R. Grange, C.-L. Hsieh, D. Psaltis, Nonlinear optical properties of core-shell nanocavities for enhanced second-harmonic generation. *Phys. Rev. Lett.* **104**, 207402 (2010).

36. T. Brabec, F. Krausz, Intense few-cycle laser fields: Frontiers of nonlinear optics. *Rev. Mod. Phys.* **72**, 545–591 (2000).

37. R. J. Tran, K. L. Sly, J. C. Conboy, Applications of surface second harmonic generation in biological sensing. *Annu. Rev. Anal. Chem.* **10**, 387–414 (2017).

38. D. S. James, P. J. Campagnola, Recent advancements in optical harmonic generation microscopy: Applications and perspectives. *BME Front.* **2021**, 3973857 (2021).

39. J. Li, J. Lu, A. Chew, S. Han, J. Li, Y. Wu, H. Wang, S. Ghimire, Z. Chang, Attosecond science based on high harmonic generation from gases and solids. *Nat. Commun.* **11**, 2748 (2020).

40. K. Hennessy, C. Högerle, E. Hu, A. Badolato, A. Imamoğlu, Tuning photonic nanocavities by atomic force microscope nano-oxidation. *Appl. Phys. Lett.* **89**, 041118 (2006).

41. M. Choi, A. Alù, A. Majumdar, Observation of photonic chiral flatbands. *Phys. Rev. Lett.* **134**, 103801 (2025).

42. M. V. Gorkunov, A. A. Antonov, V. R. Tuz, A. S. Kupriianov, Y. S. Kivshar, Bound states in the continuum underpin near-lossless maximum chirality in dielectric metasurfaces. *Adv. Opt. Mater.* **9**, 2100797 (2021).

43. L. Kühner, F. J. Wendisch, A. A. Antonov, J. Bürger, L. Hüttenhofer, Unlocking the out-of-plane dimension for photonic bound states in the continuum to achieve maximum optical chirality. *Light Sci. Appl.* **12**, 250 (2023).

44. T. Weber, L. Kühner, L. Sortino, A. Ben Mhenni, N. P. Wilson, J. Kühne, J. J. Finley, S. A. Maier, A. Tittl, Intrinsic strong light-matter coupling with self-hybridized bound states in the continuum in van der Waals metasurfaces. *Nat. Mater.* **22**, 970–976 (2023).

45. N. Bernhardt, K. Koshelev, S. J. White, K. W. C. Meng, J. E. Froch, S. Kim, T. T. Tran, D.-Y. Choi, Y. Kivshar, A. S. Solntsev, Quasi-BIC resonant enhancement of second-harmonic generation in WS_2 monolayers. *Nano Lett.* **20**, 5309–5314 (2020).

46. E. U. Condon, Theories of optical rotatory power. *Rev. Mod. Phys.* **9**, 432–457 (1937).

47. M. Jeong, J. Lee, S. Kim, X. Gong, R. Fang, Y. Yang, S. H. Chae, J. Rho, Obtuse-angled separation of chiral resonances with planar asymmetry–induced tunability of quality factors. *Sci. Adv.* **11**, eadu4875 (2025).

48. L. Nan, A. Mancini, T. Weber, G. L. Seah, E. Cortés, A. Tittl, S. A. Maier, Angular dispersion suppression in deeply subwavelength phonon polariton bound states in the continuum metasurfaces. *Nat. Photonics* **19**, 615–623 (2025).

49. S. Cao, H. Dong, J. He, E. Forsberg, Y. Jin, S. He, Normal-incidence-excited strong coupling between excitons and symmetry-protected quasi-bound states in the continuum in silicon nitride–WS_2 heterostructures at room temperature. *J. Phys. Chem. Lett.* **11**, 4631–4638 (2020).

50. E. Plum, N. I. Zheludev, Chiral mirrors. *Appl. Phys. Lett.* **106**, 221901 (2015).

51. V. Fedotov, A. Rogacheva, N. Zheludev, P. Mladyonov, S. Prosvirnin, Mirror that does not change the phase of reflected waves. *Appl. Phys. Lett.* **88**, 091119 (2006).

52. J. Kim, E. G. Carnemolla, C. DeVault, A. M. Shaltout, D. Faccio, V. M. Shalaev, A. V. Kildishev, M. Ferrera, A. Boltasseva, Dynamic control of nanocavities with tunable metal oxides. *Nano Lett.* **18**, 740–746 (2018).

53. X. He, J. Tang, H. Hu, J. Shi, Z. Guan, S. Zhang, H. Xu, Electrically driven highly tunable cavity plasmons. *ACS Photonics* **6**, 823–829 (2019).

54. C. Chakraborty, N. Vamivakas, D. Englund, Advances in quantum light emission from 2D materials. *Nanophotonics* **8**, 2017–2032 (2019).

55. E. Garmire, Nonlinear optics in daily life. *Opt. Express* **21**, 30532–30544 (2013).

56. K. Stankov, J. Jethwa, A new mode-locking technique using a nonlinear mirror. *Opt. Commun.* **66**, 41–46 (1988).

57. F. Shen, Z. Zhang, Y. Zhou, J. Ma, K. Chen, H. Chen, S. Wang, J. Xu, Z. Chen, Transition metal dichalcogenide metaphotonic and self-coupled polaritonic platform grown by chemical vapor deposition. *Nat. Commun.* **13**, 5597 (2022).

58. U. Keller, Recent developments in compact ultrafast lasers. *Nature* **424**, 831–838 (2003).

59. B. Munkhbat, P. Wróbel, T. J. Antosiewicz, T. O. Shegai, Optical constants of several multilayer transition metal dichalcogenides measured by spectroscopic ellipsometry in the 300–1700 nm range: High index, anisotropy, and hyperbolicity. *ACS Photonics* **9**, 2398–2407 (2022).

60. S. Kim, S.-C. An, Y. Kim, Y. S. Shin, A. A. Antonov, I. C. Seo, B. H. Woo, Y. Lim, M. V. Gorkunov, Y. S. Kivshar, J. I. Kim, Y. C. Jun, Chiral electroluminescence from thin-film perovskite metacavities. *Sci. Adv.* **9**, eadh0414 (2023).

61. A. B. Evlyukhin, T. Fischer, C. Reinhardt, B. N. Chichkov, Optical theorem and multipole scattering of light by arbitrarily shaped nanoparticles. *Phys. Rev. B* **94**, 205434 (2016).

62. J. Biechteler, C. Heimig, T. Weber, D. Gryb, L. Sortino, S. A. Maier, L. Menezes, A. Tittl, Fabrication optimization of van der Waals Metasurfaces: Inverse patterning boosts resonance quality factor. *Opt. Mater.* **13**, 2500920 (2025).

63. S. Fan, W. Suh, J. D. Joannopoulos, Temporal coupled-mode theory for the Fano resonance in optical resonators. *J. Opt. Soc. Am. A* **20**, 569–572 (2003).
